# GPR-GDMI: A Geometrical Dimension Detection and Morphological Inversion Method of Structural Cracks in Semi-Rigid Asphalt Pavements with Ground Penetrating Radar

**DOI:** 10.3390/s26175527

**Published:** 2026-08-31

**Authors:** Haochuan Zhou, Fanwen Meng, Jiaqi Li, Weiguang Zhang, Zheng Tong

**Affiliations:** 1 School of Transportation, Southeast University, Tianjin 300461, China; 2 Tianjin Port Engineering Institute Co., Ltd. of CCCC First Harbor Engineering Co., Ltd., Tianjin 300461, China; 3 CCCC First Harbor Engineering Co., Ltd., Tianjin 300461, China

**Keywords:** ground penetrating radar, structural cracks, geometrical dimension detection, morphological inversion, neural network, semi-rigid asphalt pavements

## Abstract

Structural cracks that develop within semi-rigid asphalt pavement structures may remain concealed beneath the pavement surface, making their opening widths, depths, and morphologies difficult to determine nondestructively. Although ground-penetrating radar (GPR) can localize subsurface reflective cracks, the hyperbolic anomalies in a B-scan, a two-dimensional cross-sectional radar profile obtained from sequential measurements along a survey line, do not directly represent the actual geometrical dimensions of the cracks. To solve this problem, this paper proposes a geometrical dimension detection and morphological inversion (GDMI) method, called the GPR-GDMI. The GPR-GDMI comprises two independently trained networks: GPR-GCDNet for geometrical dimensions detection and GPR-CMIGAN for morphology inversion. GPR-GCDNet utilizes trapezoidal boxes to represent crack dimensions in B-scan, enhanced by directional crack attention (DCA) for hyperbolic pattern extraction, a SetTrap Head for trapezoidal proposal generation, and multi-scale feature fusion for improved receptive field and coverage. GPR-CMIGAN is an unsupervised framework consisting of two independently designed U-Net generators and discriminators, with Gaussian noise injected to prevent mode collapse. The crack-dimension-constrained cycle-consistency loss incorporates GPR-GCDNet’s detected dimensions as physical constraints in the inversion, while Wasserstein loss mitigates over-constraints and improves stability during training. On the test set, GPR-GCDNet achieved an AP of 0.84, an F1-score of 0.89, and an mIoU of 0.74 under an IoU threshold of 0.5. GPR-CMIGAN achieved a performance on MS-SSIM, LPIPS, and FID of 0.9984, 0.0566, and 2.3145, respectively. Ablation studies confirm the contribution of each component. By quantitative experiments, GPR-GDMI achieves accurate detection of crack geometrical dimensions and morphological inversion based on field-collected GPR data.

## 1. Introduction

Structural cracks in semi-rigid asphalt pavements are defined as fractures embedded within the internal layers of road structures, which cannot be directly observed from the pavement surface. If not detected and addressed in time, these cracks can significantly impair the service performance and durability of the highway [1,2]. The geometrical dimensions and morphological characteristics of structural cracks are critically important for highway maintenance. For example, the depth of cracks can guide the selection of appropriate grouting techniques and the estimation of the required grouting volume.

Ground-penetrating radar (GPR) has been adopted as the principal technique for detecting structural cracks. By exploiting the propagation characteristics of high-frequency electromagnetic waves, detection is achieved through the analysis of signal differences between the crack and surrounding areas. However, diffracted waves generated at crack boundaries and reflected waves caused by structural variations propagate spherically through the medium, resulting in interference among the wave fronts. This interference ultimately produces hyperbolic anomalies in B-scan [3]. It should be noted that the dimensions of these hyperbolas do not correspond directly to the actual physical size of the cracks, as an implicit mapping relationship exists between them. Due to the limited GPR equipment, along with the influence of structural complexity and environmental noise, the construction of this mapping relationship remains challenging. As a result, the accurate extraction of the true geometrical dimensions of structural cracks remains difficult.

Before the widespread application of deep learning, conventional GPR-based geometrical characterization primarily relied on travel-time analysis, signal-attribute interpretation, analytical curve fitting, and image processing. Crack or target depth is commonly estimated from the two-way travel time using an assumed or independently measured electromagnetic-wave velocity or relative permittivity [4]. In international studies, Shihab and Al-Nuaimy [5] fitted theoretical hyperbolic signatures to radargrams to estimate the depth and radius of regular cylindrical targets. Ristic et al. [6] subsequently developed a method for jointly estimating target radius and wave-propagation velocity. For pavement cracks, Eskandari Torbaghan et al. [7] combined singular-value decomposition, median filtering, and post-processing to detect cracks from GPR responses, although the controlled experiments indicated a detectable-width threshold of approximately 1.3 mm. Rasol et al. [8] combined laboratory GPR measurements with computational modeling to identify early cracking in cement concrete pavements. Studies by Chinese research groups have focused more directly on pavement-crack attributes. Guo et al. [9] used variations in direct, diffracted, and reflected-wave amplitudes to identify the top and bottom positions of fatigue and reflective cracks and to qualitatively compare their widths. Their subsequent study related early-time-signal amplitude changes to crack width, location, and orientation [10]. Zhang et al. [11] further developed a multi-trace confidence-based relationship for estimating the dimensions of internal pavement cracks.

Although these conventional methods have clear physical interpretations, their applicability is constrained by several assumptions. Travel-time-based depth estimation requires an accurate electromagnetic-wave velocity or dielectric constant, which may vary with pavement material, moisture, temperature, and structural layer. Amplitude-based width estimation is sensitive to crack filling conditions, antenna characteristics, crack orientation, material heterogeneity, attenuation, and background clutter. Hyperbola-fitting methods generally assume regular target geometry, a homogeneous propagation medium, and a clearly identifiable diffraction signature; consequently, they cannot be directly transferred to irregular cracks in multilayered pavements. Moreover, many existing approaches provide only crack detection, approximate location, or a small number of scalar dimensions rather than complete crack morphology.

At present, numerous researchers have made extensive attempts in the field of GPR morphology reconstruction, among which GPR inversion is recognized as the key to addressing this challenge. Inversion refers to the process of recovering or estimating model parameters from observed data, constructing a mapping relationship between GPR data and the true distribution of medium. Inversion can be broadly categorized into electromagnetic physics-based inversion and neural network-based inversion approaches by principles. The former involves the formulation of partial differential equations (PDEs) that describe the relationship between electromagnetic observation parameters and the target medium properties, based on the laws governing electromagnetic wave propagation. Owing to the well-established physical foundations of this method, a wide range of inversion targets can be addressed. These include not only conventional parameters such as dielectric permittivity and electrical resistivity, but also specific features such as pipeline radius [4,5,12], volumetric water content [13], and clay content [14,15]. In general, analytical solutions to electromagnetic PDEs do not exist. Consequently, inversion must be performed by constructing an error function and solving it numerically. Commonly employed numerical techniques include Bayesian probabilistic iteration [16], Finite-Difference Time-Domain methods [17], and Full Waveform Inversion [18]. In addition, heuristic algorithms such as the Newton iteration method [19], Particle Swarm Optimization (PSO) [20,21], and Monte Carlo methods [22] are also frequently applied to solve the associated inverse problems. Despite their physical interpretability and spatial reconstruction capability, full-waveform methods remain computationally demanding and highly nonlinear. They are sensitive to the initial model, source-wavelet estimation, regularization parameters, dielectric heterogeneity, and measurement noise, and they may converge to local minima because of cycle skipping. These limitations make rapid and reliable reconstruction of irregular reflective cracks from large-scale field GPR surveys difficult.

In recent years, with the widespread adoption of deep learning methods, an increasing number of researchers have employed deep learning to address the problem of GPR inversion. Neural networks possess the capability to autonomously learn complex mapping relationships from large datasets. This not only broadens the applicable scenarios of GPR inversion but also significantly enhances computational efficiency, thereby making inversion feasible in engineering practice. The application of neural networks to inversion can be categorized into two main approaches: supervised learning-based inversion and geometry-informed neural networks for solving electromagnetic equations. The supervised learning approach requires the construction of corresponding ground-truth labels for each data instance to guide the learning process of the network. Common strategies include transforming the inversion task into an object detection problem to extract key inversion parameters [22]; alternatively, some studies have transformed inversion as a specialized semantic segmentation task, where modified segmentation networks are employed to infer the spatial distribution of subsurface materials [23,24,25]. Another technical pathway involves the use of a generative adversarial network (GAN) [26,27,28], which reformulates the inversion problem as an image-to-image translation task. In addition to enabling neural networks to complete the entire inversion process, neural networks themselves have been demonstrated as effective solvers. When applied to the solution of electromagnetic partial differential equations, they offer a means to overcome the local optima traps often encountered by conventional numerical solvers. Compared to purely data-driven inversion using neural networks, this approach exhibits physical interpretability [29].

In practical engineering, the acquisition of labeled data incurs substantial costs, as it typically requires destructive sampling of road structures. As a result, the large-scale construction of supervised learning datasets is exceedingly difficult. Currently, datasets employed in various inversion methods can be broadly classified into three categories. Aside from a small portion of field-collected data, most datasets used in existing studies are derived either from forward modeling or experimental setups. Forward model-generated datasets refer to data obtained through numerical simulations using forward modeling software such as gprMAX 4.0 or COMSOL 6.0 [30,31,32,33]. This approach allows for the construction of supervised datasets with complex and precisely defined labels. Experimentally constructed datasets are obtained by manually building simplified detection scenarios and acquiring GPR data through measurement. For instance, Dai et al. [34] buried regular metal boxes in a sand pit, while Ge et al. [28] acquired radar signals by embedding water-filled buckets in farmland. However, both types of datasets present limitations in practical engineering applications. Data generated via forward modeling are idealized, exhibiting significant differences from practical engineering. Such models typically fail to account for the complexity of real-world conditions, and the accuracy of their simulated results remains to be validated. Meanwhile, experimentally constructed datasets are generally restricted to simplified scenarios, often relying on easily manipulated materials such as sand to build experimental environments. The range of conditions that can be simulated is thus limited, making it difficult to replicate real-world complexities. Consequently, increasing attention within the GPR inversion is being directed toward the development of inversion methods based on field-collected data.

The objective of this study is to extract the crack opening width and depth information of structural cracks from field-collected GPR data and to perform a morphological inversion. A field-collected data-driven method for geometrical dimensions detection and morphological inversion of structural cracks, called GPR-GDMI, has been developed. This method consists of two main components: (1) a GPR Geometrical Crack Detection Network (GPR-GCDNet), which performs structural crack size estimation based on a forward-mapping equation and supervised learning; and (2) a GPR Crack Morphological Inversion GAN (GPR-CMIGAN), which conducts morphological inversion of structural cracks through unsupervised learning, utilizing CT scans of real core samples. A dataset required for training GPR-GCDNet is constructed based on the forward-mapping equations. Once trained, GPR-GCDNet can detect the geometrical dimensions of structural cracks directly from B-scan. Subsequently, GPR-CMIGAN, trained on unpaired B-scan and CT scan datasets, receives the outputs of GPR-GCDNet as input and performs morphological inversion of the cracks. The innovations and primary contributions of this method are summarized as follows:

(1) A method based on field-collected GPR data has been developed, enabling the detection of structural crack geometrical dimensions and the inversion of morphology.

(2) GPR-GCDNet, which was specifically designed for the characteristics of B-scan data and irregular crack-shaped labels, achieved an AP of 0.84, an F1-score of 0.89, and a mIoU of 0.74—surpassing the performance of conventional mainstream detection methods on this task.

(3) GPR-CMIGAN, constructed based on unpaired B-scan and CT scan data and incorporating crack-dimension-constrained cycle-consistency loss, enabled the inversion of crack morphology, demonstrating superior performance in MS-SSIM, LPIPS, FID, NRMSE, and PSNR.

Section 2 provides a detailed description of the network architectures and methodologies of GPR-GCDNet and GPR-CMIGAN. Section 3 outlines the procedures for data acquisition and dataset construction. Section 4 presents the results obtained from both networks. Finally, Section 5 concludes the paper.

## 2. Methodology

### 2.1. Overview

**Figure 1:**
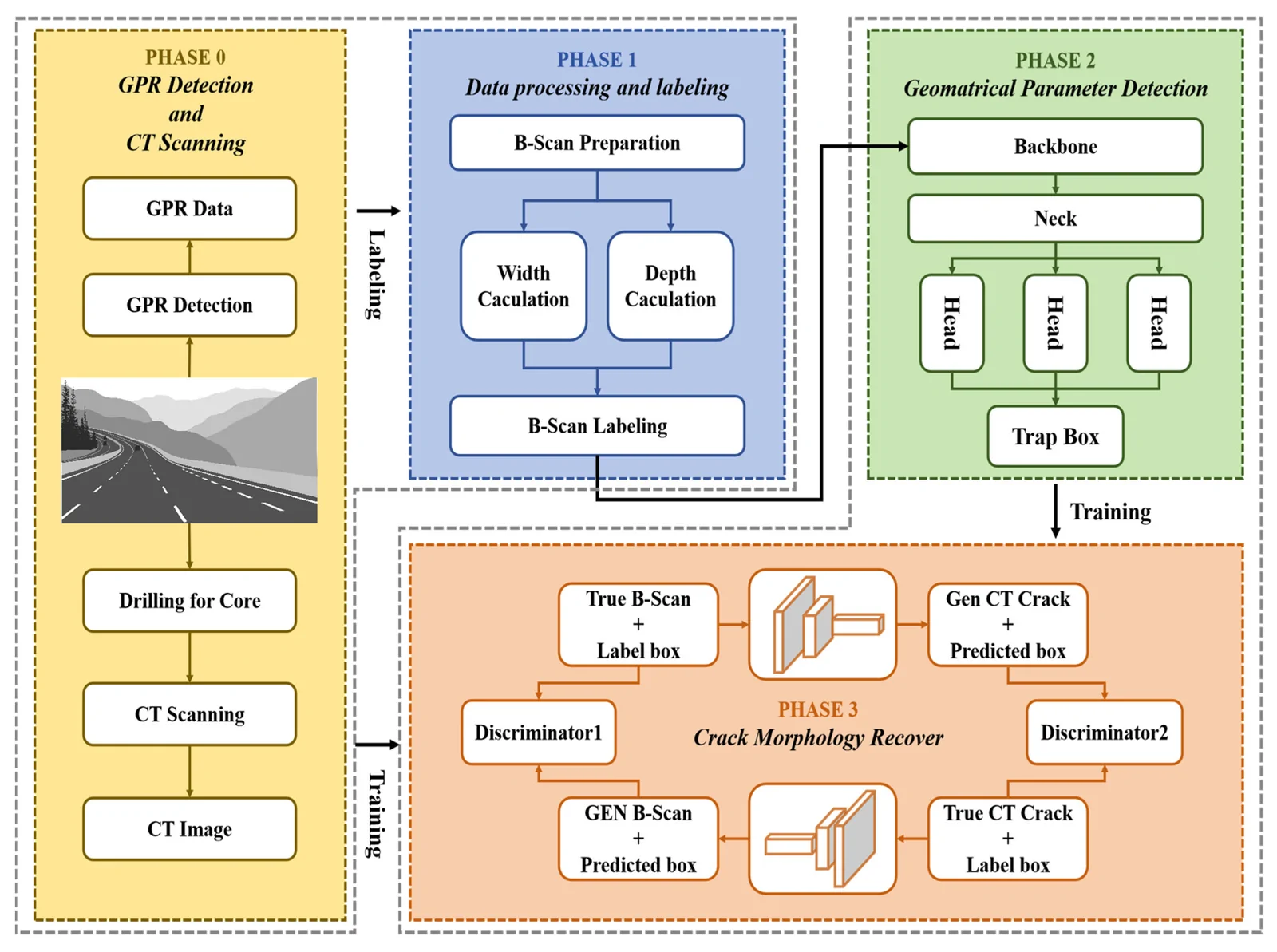
Overall workflow of GPR-GDMI.

In this study, a structural crack geometrical dimensions detection network, termed GPR-GCDNet, was first developed based on GPR-acquired B-scan and forward-mapping equations. This network is designed to extract trapezoidal boxes representing the upper and lower crack opening widths and the crack depth. Subsequently, a structural crack morphological inversion network, termed GPR-CMIGAN, was proposed. This network is constructed based on CT scans of actual crack core samples and trained using an unsupervised learning approach. Through a generative adversarial strategy, the network learns the implicit relationship between B-scan and the actual crack morphology, thereby enabling the inversion of crack shape. The overall GPR-GDMI workflow is illustrated in Figure 1, where the detailed steps of Phase 0 and Phase 1 are described in Section 3. The architectural designs of GPR-GCDNet and GPR-CMIGAN are presented first in this section.

### 2.2. GPR Geometrical Crack Detection Network

#### 2.2.1. Backbone of GPR-GCDNet

**Figure 2:**
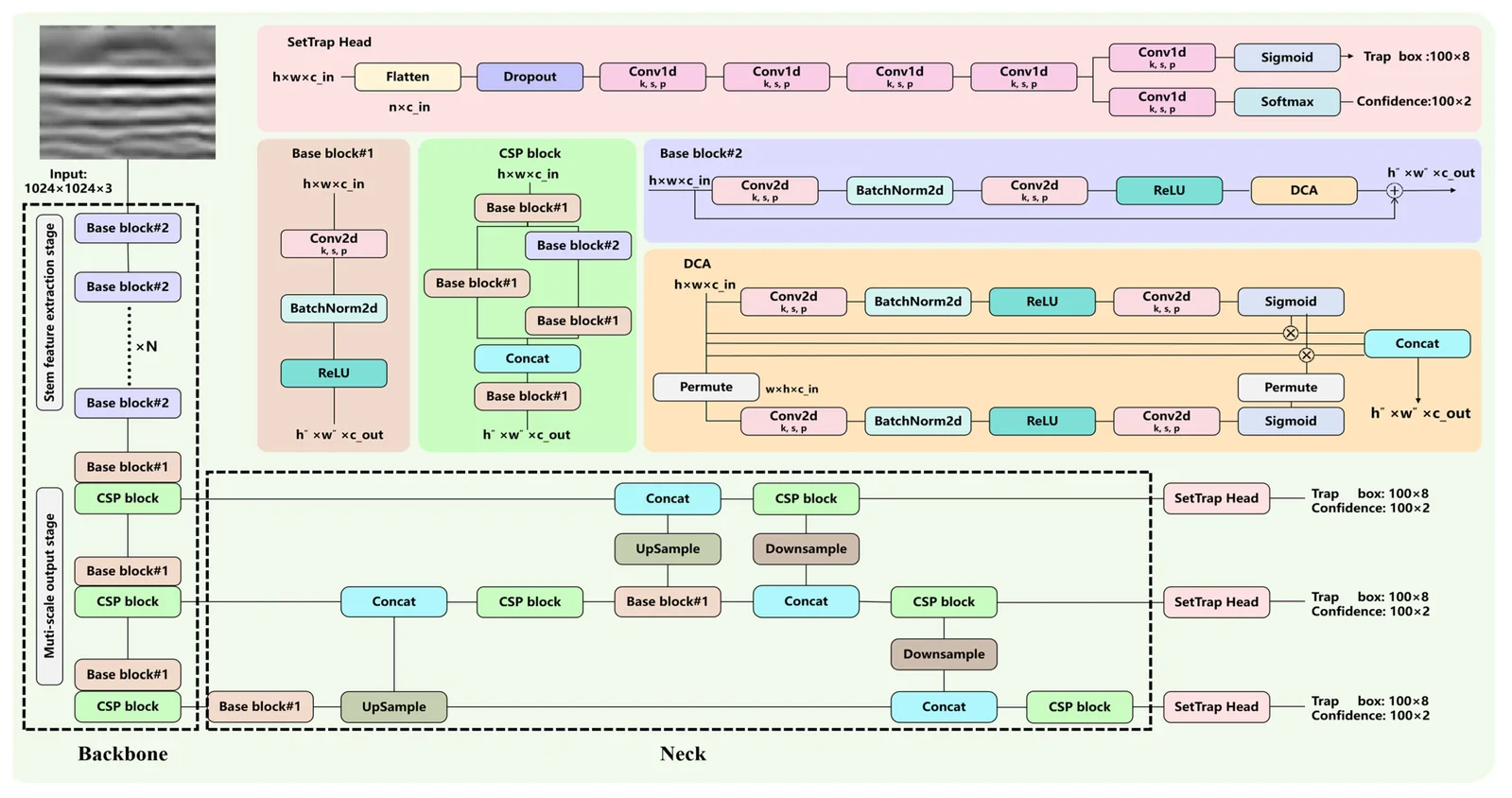
Architecture of GPR-GCDNET.

The backbone network is designed to extract features of structural cracks from B-scan through a series of convolutional layers, thereby providing enough information for the detection head. Within the backbone, an adaptive attention mechanism, termed Directional Crack Attention (DCA), is specifically designed to address the characteristics of hyperbolic regions associated with structural cracks and the geometric properties of trapezoidal boxes. The backbone of GPR-GCDNet is composed of Base block#1, Base block#2, and a CSP block connected in sequence. The detailed network architecture is illustrated in Figure 2.

According to the distribution pattern of the labels, the trapezoidal boxes occupy only approximately 3–5% of the area in B-scan. Moreover, due to the crack width being significantly smaller than its depth, the sensitivity of the labels along the *x*-axis is considerably higher than that along the *y*-axis. To increase the model’s focus on crack regions, DCA is proposed in this study. It is designed to extract adaptive weights along both the x and y directions from the feature maps, thereby enhancing the feature responses within crack regions.

The orange block in Figure 2 illustrates the structure of the DCA. The input feature map is duplicated, and one of the copies undergoes a transposition of the X and Y axes. Both resulting tensors are processed through convolutional layers to adjust the dimensions of the feature, followed by batch normalization, which is applied to integrate the global data distribution. Afterward, ReLU is employed, and the feature dimensions are further refined through an additional convolution layer. Finally, a sigmoid activation function is applied to generate two directional attention weight tensors, denoted as $\omega_{x}$ and $\omega_{y}$. This process can be expressed as:

(1) $$F_{i_{T}}^{T}=Permute\left(F_{i_{T}}\right)$$

(2) $$\omega_{x}=Sigmoid(Conv(ReLU(BN(Conv(F_{i_{T}})))))$$

(3) $$\omega_{y}=Permute(Sigmoid(Conv(ReLU(BN(Conv(F_{i_{T}}^{T}))))))$$

where, $F_{i_{T}}$ is the input feature map.

Subsequently, element-wise multiplication is performed between the two directional weight tensors to obtain a global attention tensor, which enhances the model’s overall focus on target regions. In addition, due to the higher sensitivity of trapezoidal boxes along the *x*-axis, the $\omega_{x}$ is independently multiplied with the input feature map to further strengthen the representation of horizontal features. Finally, the enhanced features obtained from the above operations are concatenated with the original input tensor and the fused feature map $F_{o_{T}}$ is output from DCA

(4) $$F_{o_{T}}=DCA\left(F_{i_{T}}\right)=Concat\left(F_{i_{T}},F_{i_{T}}\times \left(\omega_{x}\times \omega_{y}\right),F_{i_{T}}\times \omega_{y}\right)$$

The backbone architecture of GPR-GCDNet is illustrated in Figure 2. Two fundamental convolutional units are designed, called Base block#1 and Base block#2, which correspond to the brown and purple blocks in the figure. Base block#1 adopts a relatively simple structure and is primarily utilized for feature map dimension adjustment and feature fusion. Base block#2 is designed based on residual connection and incorporates not only basic components such as convolutional layers, batch normalization, and activation functions, but also integrates DCA to provide attention. The backbone can be divided into two stages: the stem feature extraction stage and the multi-scale output stage.

In the stem feature extraction stage, the input B-scan is processed through several even-numbered repetitions of Base block#2. In the first half of this stage, channel dimensionality is progressively expanded and transforms the original channels into a high-dimensional feature space, with the number of channels doubling in each layer. In the second half, the dimension of the tensor is gradually reduced to 256 × 256, while the number of channels is concurrently decreased, ultimately reaching 128 channels. This stage is primarily intended to extract features from B-scan through stacked and designed convolutional blocks.

To obtain multi-scale features, the proposed model employs CSP blocks to progressively transform the high-resolution features extracted from the backbone into low-resolution representations [35]. The input feature map to each CSP block is equally divided into two branches: one branch is directly passed to the next stage after being processed by Base block#1, while the other branch is sequentially processed through Base block#1 followed by Base block#2. The outputs of the two branches are then concatenated along the channel dimension, forming a newly fused feature map. This design integrates the concepts of residual connections and multi-granularity feature fusion, enabling the efficient integration of shallow and deep features [36]. It preserves the detailed information of the original feature maps while simultaneously enriching them with deeper semantic representations, thereby enhancing the model’s capability to represent targets at different scales. The multi-scale output stage is composed of three sequential blocks: Base block#1 and CSP block. In this stage, Base block#1 is responsible for feature dimension adjustment, while the CSP block extracts low-resolution features while retaining the structural information from high-resolution features. Each submodule outputs one feature map, and the dimensions as: $F_{o\_1}\in R^{128\times 128\times 128}$, $F_{o\_2}\in R^{256\times 64\times 64}$, $F_{o\_3}\in R^{512\times 32\times 32}$.

Finally, the three-scale feature maps are fed into the Neck module for fusion. Through upsampling and pooling operations, high-resolution and low-resolution features are cross-concatenated [37]. By combining the functionalities of Base Blocks and CSP Blocks, the Neck module facilitates cross-scale information complementarity, thereby enhancing both the global perception capability and the quality of feature representations within the network.

Conventional defect detection tasks typically employ bounding boxes to localize object regions. However, the present task focuses on detecting geometrical dimensions of structural cracks based on their hyperbolic patterns in B-scans. The target boxes are defined by the actual physical dimensions of the cracks, which commonly exhibit an elongated trapezoidal shape—narrow at the top and wider at the bottom. These trapezoidal regions occupy only a small fraction of the entire B-Scan, and the critical features are primarily concentrated at the top and bottom segments of the hyperbolic regions. Therefore, accurate detection requires the network to incorporate global contextual information. Traditional anchor-based object detection methods, which rely on grid-based partitioning and local region features, are insufficient for handling such complex labels and low information density regions [38]. To address this challenge, a set prediction strategy is adopted, and a detection head named SetTrap Head is specifically designed for this task. SetTrap Head receives the feature maps output by the Neck module, aggregates global information to generate a set of candidate trapezoidal boxes, and employs the Hungarian matching algorithm to establish one-to-one correspondence with the ground-truth labels. This approach effectively solves the positive sample assignment difficulties posed by the irregular structure of the annotations [39].

The network structure of the SetTrap Head is illustrated as the pink block in Figure 2. Three individual heads are employed to process feature maps. Upon receiving the feature map, each head flattens the input along both the X and Y dimensions into a one-dimensional. In this process, the original spatial dimensions are restructured to represent the number of boxes. As a result, subsequent convolutional layers are replaced with 1D convolutions. Before convolution, Dropout is applied to improve regularization. The final output consists of two parallel branches: one branch, activated by a Sigmoid, predicts the eight coordinates of the trapezoidal box; the other branch, activated by a SoftMax, predicts the class probabilities for background and target categories. During training, the Hungarian algorithm is employed to match positive and negative samples among the 300 boxes, based on a composite cost function that includes IoU, coordinate regression, and class probability. Positive samples are utilized to compute the IoU loss and regression loss, while all samples are used to calculate the classification loss.

#### 2.2.2. Loss Function of GPR-GCDNet

GPR-GCDNet adopts a composite loss function that integrates IoU loss, regression loss, and classification loss. This loss function is defined as follows:

(5) Loss GCDNet=Loss IoU+Loss Reg+Loss Cls

**IoU Loss:** It is difficult to directly calculate the area of a trapezoidal box. Therefore, the boxes are converted into mask representations, and the IoU is computed between the predicted mask ${Mask}_{pred}$ and the ground-truth mask ${Mask}_{truth}$. The IoU loss is then formulated as:

(6) $$Loss\;IoU=1-\frac{Intersection\left({Mask}_{pred},{Mask}_{truth}\right)}{Union\left({Mask}_{pred},{Mask}_{truth}\right)}$$

**Regression Loss:** This term measures the difference of coordinates between the predicted bounding box $\hat{B_{i}}$ and the ground-truth box $B_{i}$. Since the crack dimensions along the *y*-axis are significantly larger than those along the *x*-axis, errors in x-coordinates lead to a greater impact on localization accuracy. To address this, higher weights are assigned to the four x-coordinates. The regression loss is computed as:

(7) $$Loss\;Reg=\sum_{i}SmoothL1\left(\omega \left(\hat{B_{i}}-B_{i}\right)\right)$$

Classification Loss: To mitigate the issue of class imbalance between positive and negative samples, Focal Loss is employed. This enhances the model’s sensitivity to sparse positive samples. The classification loss is defined as:

(8) $$Loss\;Cls=-\alpha {\left(1-\hat{p}\right)}^{\gamma }\log\left(\hat{p}\right)$$

where $\hat{p}$ denotes the predicted probability for the true class, α is the weighting factor, and γ is the focusing parameter.

### 2.3. GPR Crack Morphological Inversion GAN (GPR-CMIGAN)

**Figure 3:**
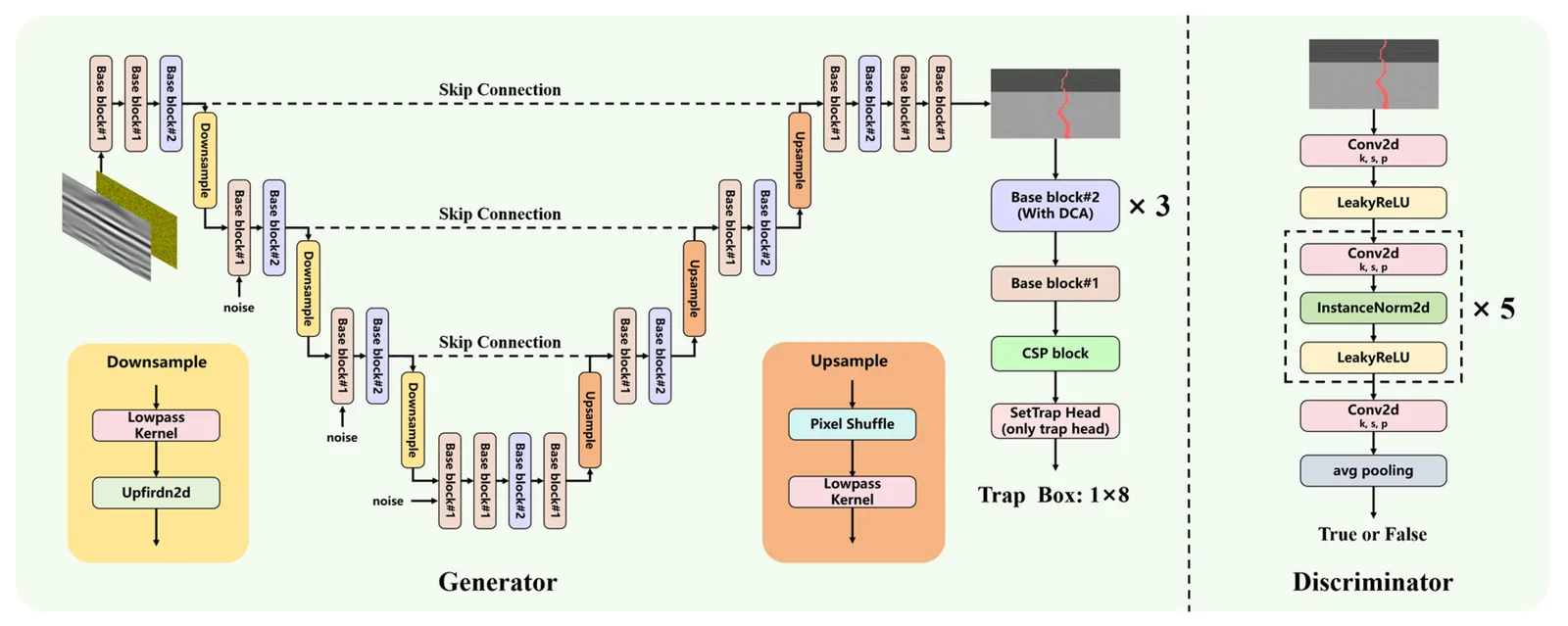
Architecture of GPR-CMIGAN.

In this study, an unsupervised generative adversarial strategy is employed to develop a crack morphological inversion network, termed GPR-CMIGAN. It is based on the geometrical dimensions detected by GPR-GCDNet and the true crack morphologies obtained from CT scans. By constructing domains of B-scan annotated with geometrical parameters and CT-based crack morphologies, and incorporating the crack-dimension-constrain cycle-consistency principle, crack morphology inversion is achieved without the need for one-to-one paired labels. GPR-CMIGAN consists of two independent generators and discriminators. On the one hand, a generative adversarial mechanism is used to enhance the realism of the generated images; on the other hand, the crack-dimension-constrain cycle-consistency principle enables the generators to mutually extract and map shared features between image domains, thereby achieving inversion. This strategy allows the model to capture the implicit mapping between the hyperbolic region of crack and true crack morphologies, facilitating the reconstruction of structural cracks [40]. To prevent the generator from mode collapse [41], random Gaussian noise is introduced. Additionally, the geometrical dimensions are employed as physical priors and embedded in the training process via a crack-dimension-constrained cycle-consistency loss, thereby enforcing geometry-informed constraints. Furthermore, Wasserstein Loss is integrated to evaluate the stylistic distance between the generated and real images from a probabilistic distribution perspective, thereby enhancing the quality of outputs. The overall network architecture is illustrated in Figure 3.

#### 2.3.1. Backbone of GPR-CMIGAN

A generator was proposed based on the U-Net architecture, equipped with an additional geometrical dimension detection branch. It shares its convolutional modules with GPR-GCDNet. To prevent the occurrence of mode collapse, random Gaussian noise was introduced both before the generator’s input of the B-scan and within the encoder stage. This noise disturbs the original probability density distribution of the input image, thereby continuously supplying the generator with disturbances. As a result, GPR-CMIGAN is encouraged to learn a more diverse set of generation modes and to enhance its generalization capability [42,43]. The mathematical formulation of this process is expressed as follows:

(9) $$F_{G}=F_{0}+G,F_{0}\in R^{x\times y\times 3}\;\;G\sim N\left(0,\sigma^{2}I\right)$$

where G is Gaussian noise.

The backbone of the generator adopts a symmetric U-shaped encoder-decoder architecture, in which features at various depths are transmitted through skip connections. The detailed structure is illustrated on the left side of Figure 3. The input B-scan is sequentially processed by several convolutional blocks shared with GPR-GCDNet, with the DCA in Base block #2 removed for this application. During the encoding stage, the feature maps are processed by downsampling, extracting four scales of features at varying depths. In the decoding stage, spatial resolution is restored through upsample blocks, and the features from the encoder are concatenated with the upsampled outputs via skip connections to enhance representational capacity. The final feature map is restored to the original spatial dimensions and channel depth to generate the inversion crack morphology. Regarding the internal structures of the downsample block and upsample block: the downsample block first applies a lowpass kernel to suppress high-frequency noise, followed by upfird2d filtering and downsampling, which reduces spatial resolution while preserving informative features. Conversely, the upsample block initially employs pixel shuffle to enlarge the feature map and subsequently applies a lowpass kernel to attenuate the noise introduced during the upsampling process [43]. The low-pass operations described above are internal components of the generator and help attenuate high-frequency fluctuations and upsampling artifacts during feature transformation. In contrast, the random Gaussian noise injected at the generator input and encoder stages is introduced for distribution perturbation and regularization to mitigate mode collapse; it should not be interpreted as preprocessing or denoising of the measured GPR signals.

The image generated by the backbone is subsequently fed into a geometrical dimension detection branch to detect the dimensions of the generated crack. This branch network is a simplified variant of GPR-GCDNet. The generated image is first processed by three Base block#2 for feature extraction. The resulting feature maps are then passed through Base block#1 followed by a CSP block for size and channel adjustment. Finally, the features are input into a SetTrap Head, which is configured to retain only the dimension detection branch, yielding a single set of crack geometrical dimensions. Within the dimension detection branch, the DCA is preserved in Base block#2 to enhance sensitivity to spatially distributed crack features. The detected geometrical dimensions are incorporated into the crack-dimension-constrained cycle-consistency loss, ensuring that the generated crack dimensions are consistent with those inferred from the original B-scan. This mechanism guides the generator to produce morphologies that preserve the original structural dimensions.

The discriminator in GPR-CMIGAN adopts a PatchGAN architecture, consisting of five convolutional modules. Each module comprises a convolutional layer, instance normalization, and a LeakyReLU activation function, which collectively enable channel expansion and progressive downsampling, while enhancing the network’s sensitivity to texture variations. The detailed network structure is shown on the right side of Figure 3. The PatchGAN architecture offers the advantage of model compactness and emphasizes localized discrimination, effectively suppressing artifacts that may arise from directional variations in crack propagation. Instance normalization contributes to improved style consistency across diverse crack morphologies. Compared with a global discriminator, this architecture involves fewer parameters and exhibits higher computational efficiency, thereby promoting stable training and enhancing the quality of generated image details in unpaired image generation conducted by GPR-CMIGAN.

#### 2.3.2. Loss Function of GPR-CMIGAN

Pixel-wise comparison strategies used to evaluate image difference often led to mode collapse in generated images, causing the network to overfit and limiting the diversity of outputs. Since the labels and input images in GPR-CMIGAN are not paired in a one-to-one correspondence, it becomes necessary to introduce a method capable of capturing stylistic differences between images.

Wasserstein loss provides a principled means of measuring the minimum “transport cost” required to shift one probability distribution into another. Compared with traditional JS divergence, Wasserstein loss more effectively mitigates issues such as gradient vanishing and mode collapse, making it more suitable for GAN training [43]. Specifically, under the Wasserstein loss framework, the objective of the generator G is to produce outputs $\tilde{x}$ that are indistinguishable by discriminator D. The corresponding mathematical formulation is:

(10) $$Loss\;G\left(D,\tilde{x}\right)={-E}_{\tilde{x}\sim p_{g}}\left[D\left(\tilde{x}\right)\right]$$

The discriminator evaluates the generated and real images by computing the score difference between them under a 1-Lipschitz constraint. To reduce the strictness imposed by this constraint, a gradient penalty term is introduced. This penalty helps to enforce the Lipschitz continuity condition more flexibly. The loss function for the discriminator is:

(11) $$Loss\;D\left(D,x,\tilde{x}\right)=E_{\tilde{x}\sim p_{g}}\left[D\left(\tilde{x}\right)\right]-E_{x\sim p_{r}}\left[D\left(x\right)\right]+\lambda E_{\tilde{x}\sim p\left(\tilde{x}\right)}\left[{\left({\left\|\nabla_{\tilde{x}}D\left(\tilde{x}\right)\right\|}_{2}-1\right)}^{2}\right]$$

The cycle-consistency principle serves as the core enabling unsupervised learning in CycleGAN. Unpaired B-scan and CT images are independently fed into the network to generate corresponding images in the alternate domain. The cycle-consistency principle constructs a closed-loop generation path between the two generators, thereby enabling mutual verification across image domains and constraining the directionality of image translation [40]. However, this mechanism does not include the geometrical dimensions of the cracks, resulting in generated crack representations that lack physical interpretability. Consequently, a distance may exist between the generated crack morphologies and the actual crack morphologies.

**Figure 4:**
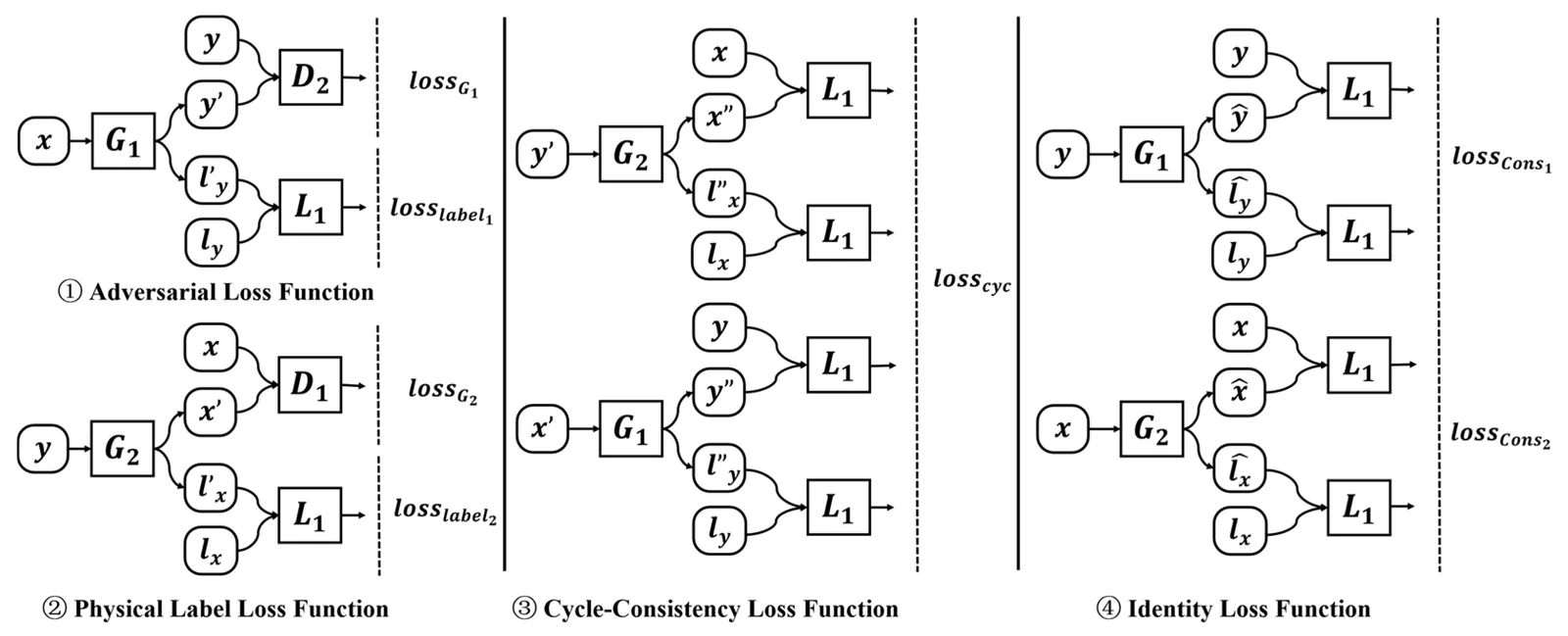
Crack-dimension-constrained cycle-consistency loss.

To increase physical constraints, a crack-dimension-constrained cycle-consistency loss is proposed in this study, wherein the geometrical dimensions of structural cracks are embedded into the training process as a constraint. Specifically, when a B-scan is fed into the generator, the resulting image produced by the inversion backbone is further input into the geometrical dimension detection branch. The predicted geometrical dimensions are compared with those originally derived from the input B-scan, and the resulting difference is used to compute a loss term. This loss is directly incorporated into the backpropagation process of the generator, thereby serving as an additional geometry-informed constraint that encourages the generated cracks to possess geometrically physical dimensions. For the generator, the crack-dimension-constrained cycle-consistency loss comprises four components, which are illustrated in Figure 4.

The adversarial loss is formulated based on the game mechanism between the generator and the discriminator, guiding the generator to progressively approximate the target image domain. To evaluate the performance of the generator, Wasserstein loss is employed.

(12) $${Loss\;G}_{1}\left(D_{1},y^{\prime }\right)={-E}_{\tilde{y^{\prime }}\sim p_{g}}\left[D_{1}\left(y^{\prime }\right)\right]$$

(13) $${Loss\;G}_{2}\left(D_{2},x^{\prime }\right)={-E}_{\tilde{x^{\prime }}\sim p_{g}}\left[D_{2}\left(x^{\prime }\right)\right]$$

where $y^{\prime }$ and $x^{\prime }$ are results of generators, and $D_{1}$ and $D_{2}$ are discriminators.

The Physical Label Loss Function is introduced to evaluate the accuracy of the coordinate detection generated by the geometrical dimension detection branch of the generator. The coordinates are compared with the corresponding ground-truth labels from the input B-scan using an L1 loss, thereby imposing a geometry-informed constraint on the generator. This loss can be formulated as:

(14) $${Loss\;label}_{1}\left(l_{x},l_{x}^{\prime }\right)={\left\|l_{x}-l_{x}^{\prime }\right\|}_{1}$$

(15) $${Loss\;label}_{2}\left(l_{y},l_{y}^{\prime }\right)={\left\|l_{y}-l_{y}^{\prime }\right\|}_{1}$$

where $l_{x}$ and $l_{y}$ are the ground-truth labels, and $l_{x}^{\prime }$ and $l_{y}^{\prime }$ are detected labels.

Cycle-Consistency Loss Function, the output generated by generator $G_{1}$ from input x is passed through generator $G_{2}$, and x is expected to be accurately reconstructed. Moreover, the geometrical dimensions detected by $G_{2}$ should match the original ground-truth parameters associated with x. The same principle applies in the reverse direction. Accordingly, the following loss function is formulated:

(16) $$\begin{array}{c} Loss\;cyc\left(x,y,x^{\prime \prime },y^{\prime \prime },l_{x},l_{y},l_{x}^{\prime \prime },l_{y}^{\prime \prime }\right)=\\ E_{x\sim p\left(x\right)}{\left\|x^{\prime \prime }-x\right\|}_{1}+E_{y\sim p\left(y\right)}{\left\|y^{\prime \prime }-y\right\|}_{1}+{\left\|l_{x}-l_{x}^{\prime \prime }\right\|}_{1}+{\left\|l_{y}-l_{y}^{\prime \prime }\right\|}_{1} \end{array}$$

where x and y are original images, $x^{\prime \prime }$ and $y^{\prime \prime }$ are the images acquired from cycle generation, and $l_{x}^{\prime \prime }$ and $l_{y}^{\prime \prime }$ are the labels acquired from cycle generation.

**Identity Loss Function:** For the generator $G_{1}$, which maps input x to output $y^{\prime }$, it is required that when y is directly input into the generator, the output remains unchanged as y. This constraint ensures consistency in the generator’s translation direction. The following loss function is formulated:

(17) $${Loss\;Cons}_{1}\left(G_{1},y\right)={\left\|G_{1}\left(y\right)-y\right\|}_{1}$$

(18) $${Loss\;Cons}_{2}\left(G_{2},x\right)={\left\|G_{2}\left(x\right)-x\right\|}_{1}$$

For both generators, the cycle-consistency principle is considered, and identical loss functions are backpropagated through $G_{1}$ and $G_{2}$, allowing the evolution directions of both generators to be jointly optimized. The final loss function for the generators is therefore defined as:

(19) $$\begin{array}{c} Loss\;Gen={Loss\;G}_{1}\left(D_{1},y^{\prime }\right)+{Loss\;G}_{2}\left(D_{2},x^{\prime }\right)+{Loss\;label}_{1}\left(l_{x},l_{x}^{\prime }\right)\\ +{Loss\;label}_{2}\left(l_{y},l_{y}^{\prime }\right)+Loss\;cyc\left(x,y,x^{\prime \prime },y^{\prime \prime },l_{x},l_{y},l_{x}^{\prime \prime },l_{y}^{\prime \prime }\right)\\ +{Loss\;Cons}_{1}\left(G_{1},y\right)+{Loss\;Cons}_{2}\left(G_{2},x\right) \end{array}$$

For the discriminators, the two networks are responsible for independent discrimination tasks and therefore require separate loss functions to be backpropagated individually. The discriminator loss in GPR-CMIGAN is formulated based on the Wasserstein loss and is expressed as follows:

(20) $${Loss\;D}_{1}\left(D_{1},x,y^{\prime }\right)=E_{y^{\prime }\sim p_{g}}\left[D_{1}\left(y^{\prime }\right)\right]-E_{x\sim p_{r}}\left[D_{1}\left(x\right)\right]+\lambda E_{y^{\prime }\sim p\left(y^{\prime }\right)}\left[{\left({\left\|\nabla_{y^{\prime }}D_{1}\left(y^{\prime }\right)\right\|}_{2}-1\right)}^{2}\right]$$

(21) $${Loss\;D}_{2}\left(D_{2},y,x^{\prime }\right)=E_{x^{\prime }\sim p_{g}}\left[D_{2}\left(x^{\prime }\right)\right]-E_{y\sim p_{r}}\left[D_{2}\left(y\right)\right]+\lambda E_{x^{\prime }\sim p\left(x^{\prime }\right)}\left[{\left({\left\|\nabla_{\tilde{y}}D_{2}\left(x^{\prime }\right)\right\|}_{2}-1\right)}^{2}\right]$$

## 3. Data Processing and Experiment Preparation

### 3.1. Acquiring of Geometrical Dimension Calculation Formula

#### 3.1.1. Width Estimation Formula of Crack

**Figure 5:**
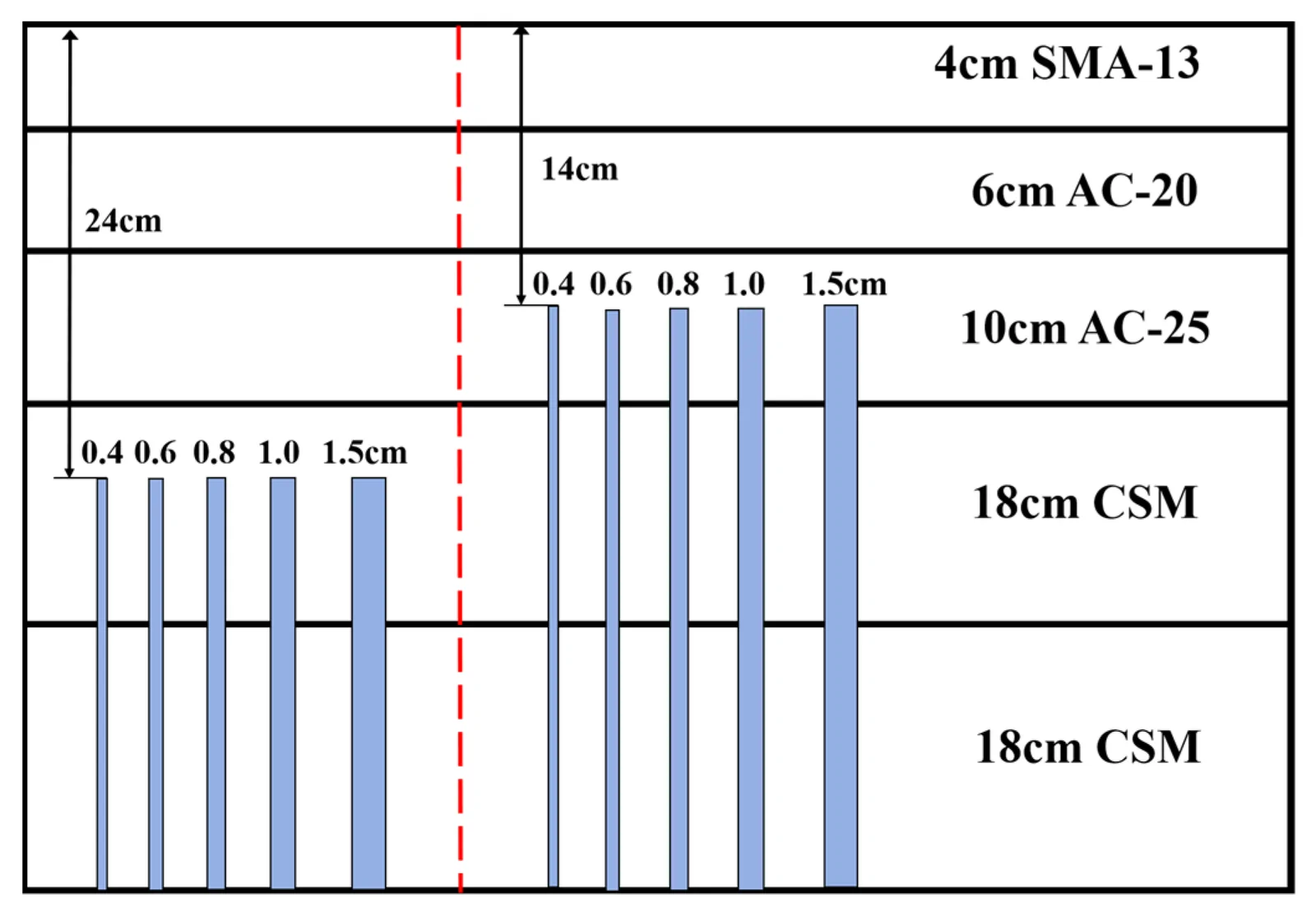
Forward Modeling Schematic Diagram.

In our previous research, a three-dimensional multiphase heterogeneous forward model was established. Specifically, coarse aggregate particles with distinct morphological characteristics were embedded across various structural layers of the pavement, and the reconstructed aggregates were incorporated into an FDTD model. Other pavement materials, such as fine aggregates, asphalt, cement mortar, and air, were modeled as randomly distributed media based on volume fraction, thereby forming a complete representation of the pavement structure. Detailed modeling procedures are referred to the publications of the research team [11]. Reflective cracking is the most common type of structural cracking observed in semi-rigid asphalt pavements. Based on the characteristics of the collected data, two types of reflective cracks were simulated: one in which the crack does not penetrate the base layer, and another in which the crack penetrates the surface layer. The simulated types of reflective cracking are illustrated in Figure 5.

For the two types of reflective cracks, the burial depths of the cracks were fixed at 24 cm and 14 cm, respectively. The crack widths were set to 0.4 cm, 0.6 cm, 0.8 cm, 1.0 cm, and 1.5 cm. The relative dielectric constant of the medium within the crack was assigned values of 1, 30, and 81, corresponding to three representative crack conditions: dry, moist, and saturated, respectively. In our prior research, the relationship between crack width and the amplitude of the GPR signal was established, and the results were normalized.

When the crack reflection reaches the surface layer, the formula used to calculate the upper width of the crack is given by:

(22) $$w^{\prime }=6.5839A-0.6207\;\;\;\;\;\;\;R^{2}=0.9905\left(Dry\right)$$

(23) $$w^{\prime }=6.9837A-1.7398\;\;\;\;\;\;\;\;R^{2}=0.9818\left(Wet\right)$$

The formula used to calculate the lower width of the crack is given by:

(24) $$w^{\prime \prime }=18.076A-0.8276\;\;\;\;\;\;\;R^{2}=0.9940\left(Dry\right)$$

(25) $$w^{\prime \prime }=8.7605A-0.8111\;\;\;\;\;\;\;R^{2}=0.9830\left(Wet\right)$$

When the crack reflection does not reach the surface layer, the formula used to calculate the upper width of the crack is given by:

(26) $$w^{\prime }=2.3068A-0.4065\;\;\;\;\;\;\;\;R^{2}=0.9965\left(Dry\right)$$

(27) $$w^{\prime }=3.4264A-1.317\;\;\;\;\;\;\;\;R^{2}=0.9902\left(Wet\right)$$

The formula used to calculate the lower width of the crack is given by:

(28) $$w^{\prime \prime }=18.207A-0.8137\;\;\;\;\;\;\;\;R^{2}=0.9958\left(Dry\right)$$

(29) $$w^{\prime \prime }=3.7404A-1.013\;\;\;\;\;\;\;\;R^{2}=0.9564\left(Wet\right)$$

where A is the average amplitude of the crack signal. The values of $\varepsilon_{c}$ = 1, 30, and 81 were introduced as idealized effective-permittivity scenarios spanning air-filled, partially water-filled, and water-filled cracks, respectively. They should not be interpreted as direct measurements of the field moisture state. For regression, the ($\varepsilon_{c}$ = 1) samples were assigned to the dry branch, while the two water-bearing scenarios, $\varepsilon_{c}$ = 30 and $\varepsilon_{c}$ = 81, were pooled into the wet branch. Consequently, Equations (22)–(29) contain two operational branches rather than three separate moisture-specific regressions.

#### 3.1.2. Depth Estimation Formula of Crack

**Figure 6:**
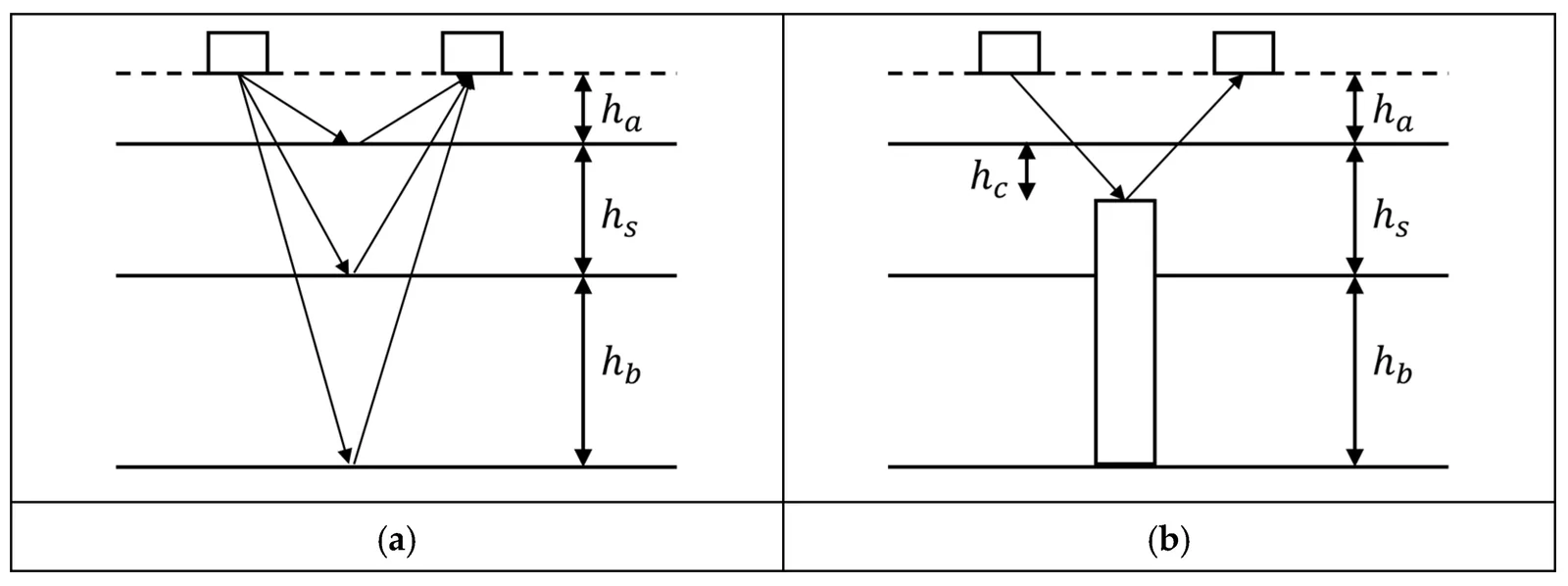
Two-way Travel Time Method Diagram. (**a**) Two-way travel of electronic waves in a pavement without cracks. (**b**) Two-way travel of electronic waves in a pavement with a crack.

The two-way travel time method is a commonly used approach in GPR for estimating the burial depth of underground targets. It can be interpreted as the propagation of electromagnetic waves along two symmetric paths from the transmitting antenna to the target, then back to the receiving antenna. The propagation path is illustrated in Figure 6.

Based on the geometrical configuration among the transmitting antenna, target, and receiving antenna, as well as the electromagnetic wave propagation equations in a layered medium, the following four relational expressions can be derived:

(30) $${\left(\frac{t_{a}}{2}\times \frac{c}{\sqrt{\varepsilon_{a}}}\right)}^{2}={\left(\frac{d}{2}\right)}^{2}+{h_{a}}^{2}$$

(31) $${\left(\frac{t_{a}}{2}\times \frac{c}{\sqrt{\varepsilon_{a}}}+\frac{t_{s}-t_{a}}{2}\times \frac{c}{\sqrt{\varepsilon_{s}}}\right)}^{2}={\left(\frac{d}{2}\right)}^{2}+{\left(h_{a}+h_{s}\right)}^{2}$$

(32) $${\left(\frac{t_{a}}{2}\times \frac{c}{\sqrt{\varepsilon_{a}}}+\frac{t_{s}-t_{a}}{2}\times \frac{c}{\sqrt{\varepsilon_{s}}}+\frac{t_{b}-t_{s}}{2}\times \frac{c}{\sqrt{\varepsilon_{b}}}\right)}^{2}={\left(\frac{d}{2}\right)}^{2}+{\left(h_{a}+h_{s}+h_{b}\right)}^{2}$$

(33) $${\left(\frac{t_{a}}{2}\times \frac{c}{\sqrt{\varepsilon_{a}}}+\frac{t_{c}-t_{a}}{2}\times \frac{c}{\sqrt{\varepsilon_{s}}}\right)}^{2}={\left(\frac{d}{2}\right)}^{2}+{\left(h_{a}+h_{c}\right)}^{2}$$

where $h_{a}$ denotes the height of the antenna above the ground, $h_{s}$ is the thickness of the surface layer, $h_{b}$ is the thickness of the base layer, and $h_{c}$ is the distance from the upper of the crack to the surface. d denotes the horizontal spacing between the transmitting and receiving antennas. The relative dielectric constant of air, the surface layer, and the base layer are denoted as $\varepsilon_{a}$, $\varepsilon_{s}$ and $\varepsilon_{b}$. In this study, the values of the relative dielectric constant are set as: $\varepsilon_{a}=1$, $\varepsilon_{s}=5.5$, $\varepsilon_{b}=10.5$. The antenna height $h_{a}$ is set to **0.15 m**, and the antenna spacing d is 0.1 m. Based on these parameters, the following formulas are derived for computing $h_{s}$, $h_{b}$, and $h_{c}$:

(34) $$h_{s}=\sqrt{{\left(\frac{t_{a}}{2}\times \frac{c}{\sqrt{\varepsilon_{a}}}+\frac{t_{s}-t_{a}}{2}\times \frac{c}{\sqrt{\varepsilon_{s}}}\right)}^{2}-{\left(\frac{d}{2}\right)}^{2}}-h_{a}$$

(35) $$h_{b}=\sqrt{{\left(\frac{\sqrt{d^{s}+4{h_{a}}^{2}}}{2}+\frac{t_{s}-t_{a}}{2}\times \frac{c}{\sqrt{\varepsilon_{s}}}+\frac{t_{b}-t_{s}}{2}\times \frac{c}{\sqrt{\varepsilon_{b}}}\right)}^{2}}-h_{a}$$

(36) $$h_{c}=\sqrt{{h_{a}}^{2}+\sqrt{d^{s}+4{h_{a}}^{2}}\times \frac{t_{c}-t_{a}}{2}\times \frac{c}{\sqrt{\varepsilon_{s}}}+{\left(\frac{t_{c}-t_{a}}{2}\times \frac{c}{\sqrt{\varepsilon_{s}}}\right)}^{2}}-h_{a}$$

The crack depth $D_{c}$ can then be calculated using the following equation:

(37) $$D_{c}=h_{s}+h_{b}-h_{c}$$

Equations (22)–(37) should be interpreted as calibrated forward-mapping relationships rather than universally applicable analytical solutions. The calibration domain comprises two representative reflective-crack configurations in semi-rigid asphalt pavements, crack widths of 0.4–1.5 cm, burial depths of 14 and 24 cm, and idealized crack-infill permittivities of 1, 30, and 81. The R^2^ values of 0.9564–0.9965 demonstrate the goodness of fit within this simulated domain but do not guarantee equivalent accuracy for pavement structures, antenna configurations, crack geometries, or dielectric conditions outside the calibration range. In field applications, amplitude-based width estimation may be affected by moisture, attenuation, crack orientation, material heterogeneity, and background clutter, whereas travel-time-based depth estimation remains dependent on the assumed layer permittivities. Therefore, CT measurements from field-extracted cores were used as an independent physical reference to assess the practical reliability of the calculated labels.

### 3.2. Dataset Preparation

Four different data products are used in the proposed framework. First, field-acquired GPR B-scans constitute the input data for both GPR-GCDNet and GPR-CMIGAN. Second, equation-derived geometrical labels are calculated from the GPR amplitude and travel-time information and used only as supervisory labels for GPR-GCDNet and as dimensional constraints for GPR-CMIGAN. Third, CT-derived morphologies are segmented from physical pavement cores and serve as physical references for dimensional validation and as real samples in the morphology domain of GPR-CMIGAN. Fourth, synthetic morphology images are generated by emulating the crack patterns observed in the CT images and are used only to increase the diversity of the GPR-CMIGAN morphology domain. The reconstructed morphologies produced by GPR-CMIGAN are model outputs and are not treated as training labels or physical ground truths.

#### 3.2.1. Dataset of GPR-GCDNet

**Figure 7:**
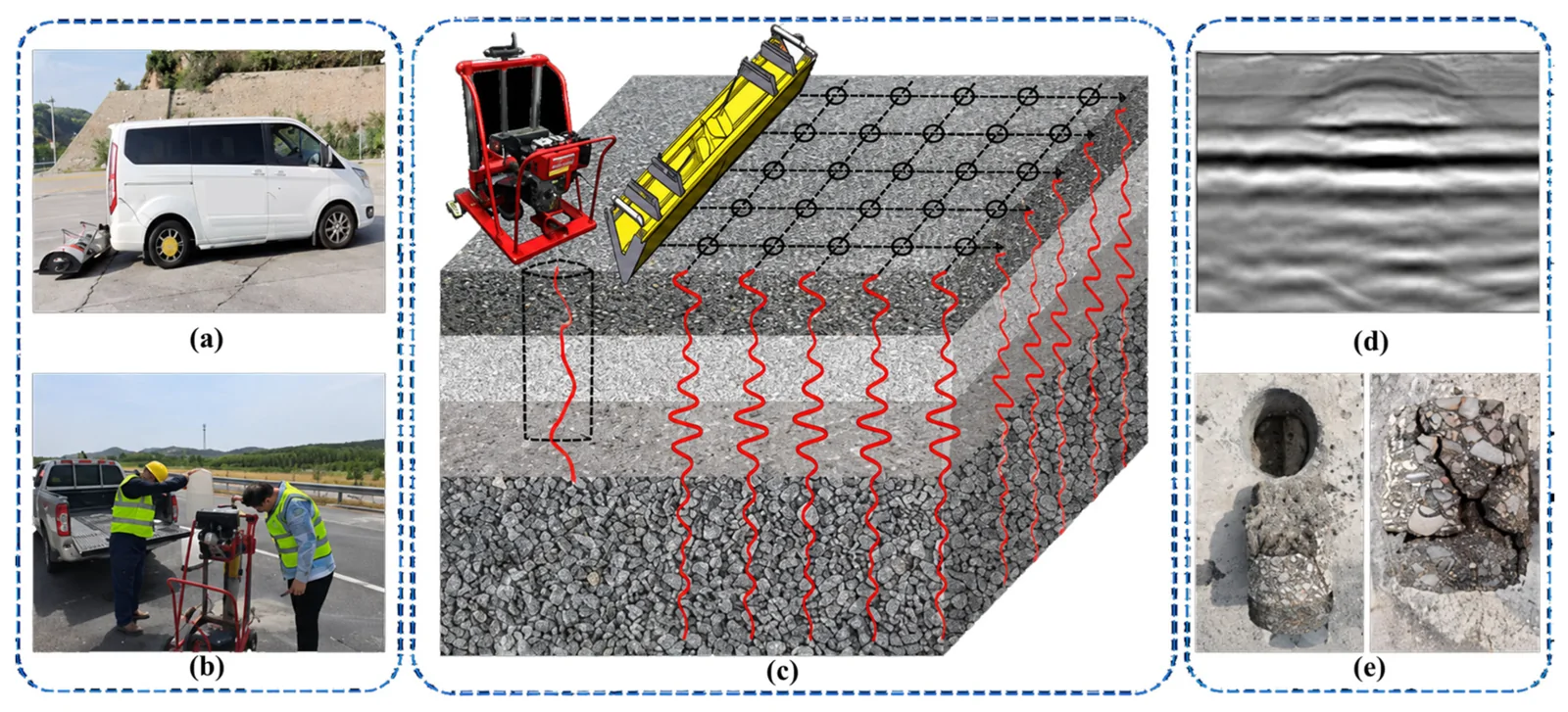
GPR data-collection workflow: (**a**) vehicle-mounted impulse GPR system used for highway scanning; (**b**) field coring at a GPR-indicated crack location; (**c**) schematic of multichannel GPR scanning and electromagnetic-wave propagation in the layered pavement structure; (**d**) representative GPR B-scan containing a crack-induced hyperbolic reflection; and (**e**) borehole and extracted cracked pavement core used for subsequent CT scanning and geometrical validation.

**Figure 8:**
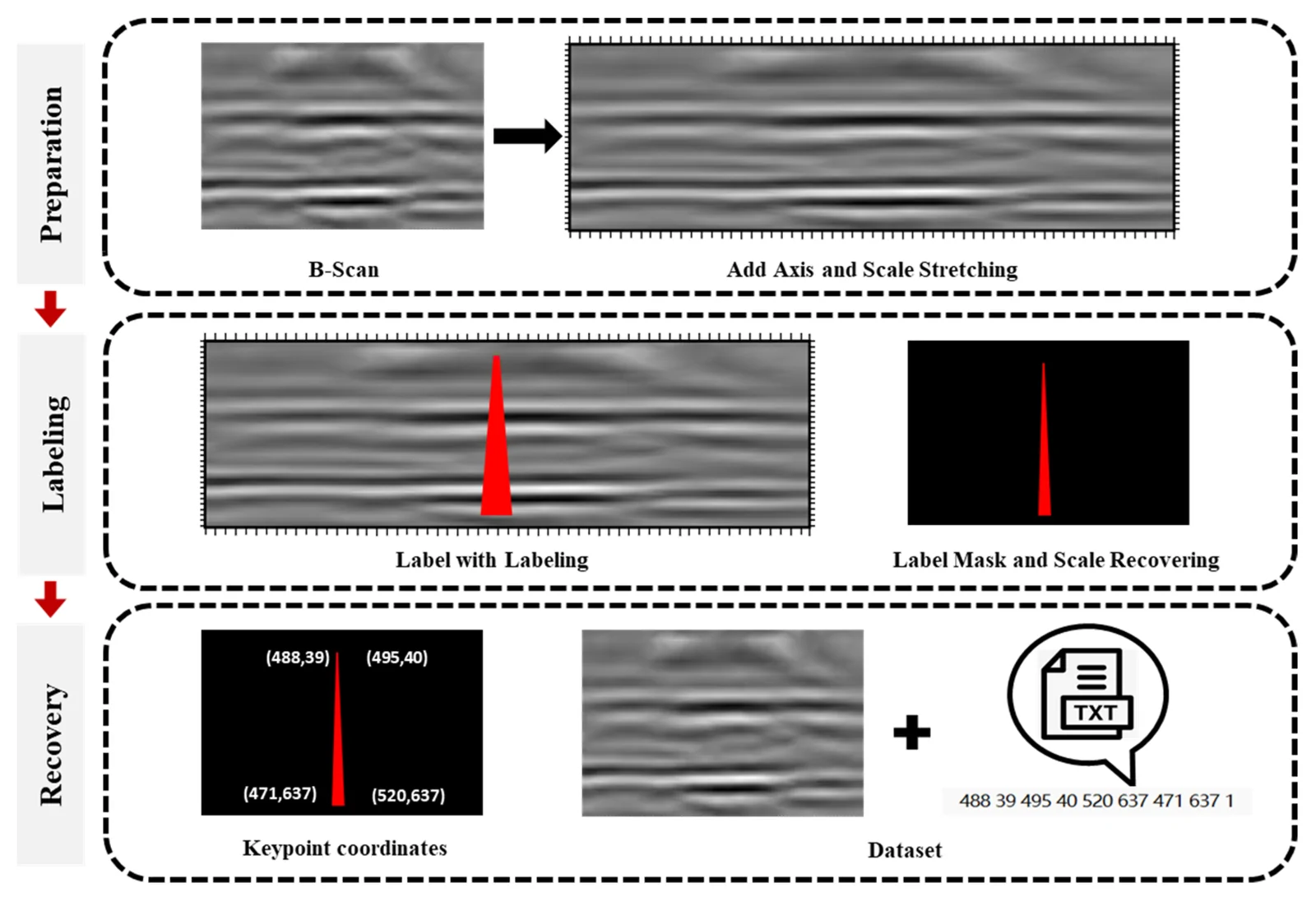
Workflow for annotating crack geometrical dimensions in GPR B-scans.

**Figure 9:**
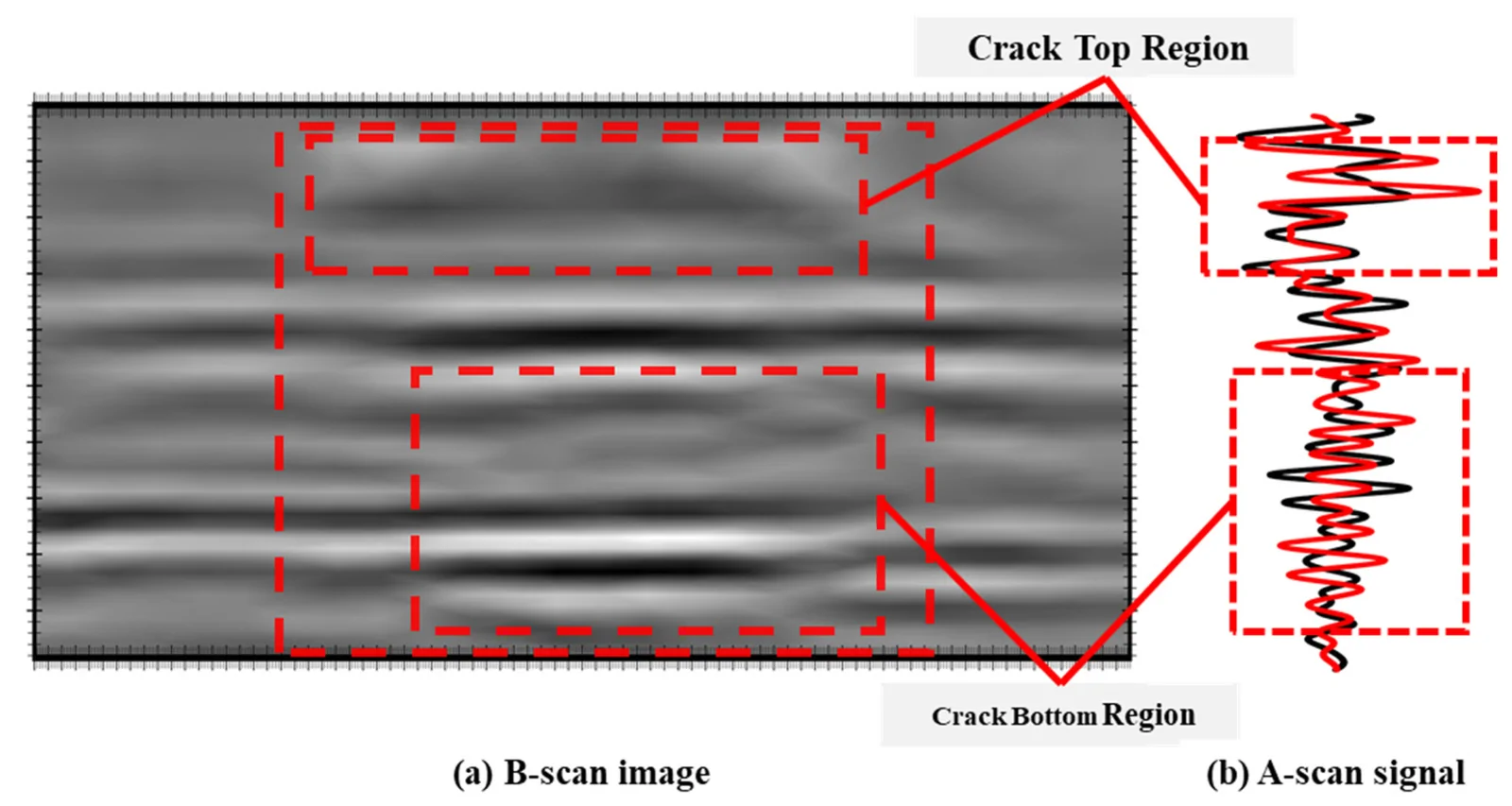
Comparison of A-Scan Between Cracked and Intact Regions.

B-scan data representing structural cracks were collected from highways in Zhejiang, Shanxi, and Anhui provinces using the Impulse GPR system, with the processing shown in Figure 7. The geometrical dimension detection model requires a B-scan dataset annotated with crack geometrical dimensions. In GPR data acquisition, an A-scan is a one-dimensional reflected signal recorded at a single antenna position, whereas a B-scan is a two-dimensional cross-sectional profile generated by arranging consecutive A-scans along the survey direction. The estimation formulas have been established in Section 3.1. In Figure 8, the annotation procedure for crack geometrical parameters in B-scan images is as follows:

Step 1: The B-scan signal intensity is first normalized, and hyperbolic anomalies indicative of cracks are identified. The upper and lower amplitude of crack A-scan signals are extracted and averaged. Then, the upper and lower crack opening widths are derived using Equations (22)–(29). Before applying Equations (22)–(29), the crack-related A-scan was phase-corrected using the reflection from a known pavement interface. The sign of the normal-incidence reflection coefficient is governed by the dielectric contrast across the crack interface. Therefore, air-filled and water-bearing cracks exhibit opposite polarity patterns because their effective dielectric constants are, respectively, lower and higher than that of the surrounding pavement. A trace was assigned to the dry branch when its first crack-related reflection was consistent with a low-permittivity infill. A polarity-reversed reflection accompanied by increased attenuation was assigned to the wet branch. When the polarity or attenuation evidence was insufficient for a reliable classification, both branches were evaluated, and the interval between the two estimates was retained as moisture-state uncertainty. Subsequently, the crack depth is calculated based on signal travel time and Equations (34)–(37). Figure 9 illustrates the comparison between A-scan signals from crack and non-crack regions.

Step 2: Based on the scaling ratio between B-scan pixel dimensions and actual physical dimensions, coordinate axes are added to the B-scan image. The original 1080 × 680 resolution image, representing a physical area of 80 cm × 50 cm, is rescaled to 2160 × 680 to facilitate annotation. Using the Labelme annotation tool, trapezoidal boxes are created based on the geometrical dimensions calculated in Step 1. These boxes are aligned with the center of the hyperbolic reflection in base layer. The annotations are saved in json files, converted into masks, and the annotated B-scan and masks are then resized back to their original resolution.

Step 3: The mask files are converted into pixel coordinate labels, which are stored in txt files to serve as the final annotations.

Field-acquired GPR B-scans inevitably contain environmental noise, background clutter, and acquisition-dependent amplitude variations. In the present study, the signal intensity of each B-scan was normalized using the same procedure before annotation and network input. After source-level dataset partitioning, additive Gaussian noise was applied only to the training subset with a probability of 20%, whereas the validation and test subsets were not subjected to noise augmentation. This strategy exposes GPR-GCDNet to perturbed inputs during training and improves its tolerance to moderate signal fluctuations. However, it is an augmentation strategy rather than a signal-recovery procedure and cannot reconstruct crack responses that are completely obscured by noise.

In total, 704 annotated samples were collected from 10 survey sections on highways in Zhejiang, Shanxi, and Anhui Provinces, China, with a ratio of about 1.2:1.0:1.4. These samples include independent structural cracks under different pavement structures and survey conditions. The dataset was divided into training, validation, and testing subsets according to a 7:2:1 ratio. To prevent information leakage, dataset partitioning was performed at the level of the original survey section and physical crack before image cropping, preprocessing, and data augmentation. All B-scans, cropped images, and augmented samples originating from the same physical crack or continuous survey section were assigned exclusively to one subset. Consequently, no sample derived from a crack represented in the training set was included in the validation or test set. The final training, validation, and test sets originated from independent physical cracks, respectively. Data augmentation was applied only to the training subset after completion of the source-level partition, including additive Gaussian noise and mirroring. Each operation was applied with a probability of 20%.

#### 3.2.2. Dataset of GPR-CMIGAN

As GPR-CMIGAN adopts an unsupervised learning strategy, two independent image domain datasets are required. The B-scan domain is constructed directly from the dataset established in Section 3.2.1. To obtain true crack morphologies and construct a dataset for morphological inversion, core sampling was conducted on the Shanxi highway, with each core having a diameter of 8 cm. The core samples were scanned using an X-ray computed tomography (CT) system. Each core was placed vertically within the scanning chamber and scanned using an axial scan protocol, with the X-ray tube voltage and current set to 200 kV and 0.28 mA, respectively. The scanning resolution was 0.2 mm.

**Figure 10:**
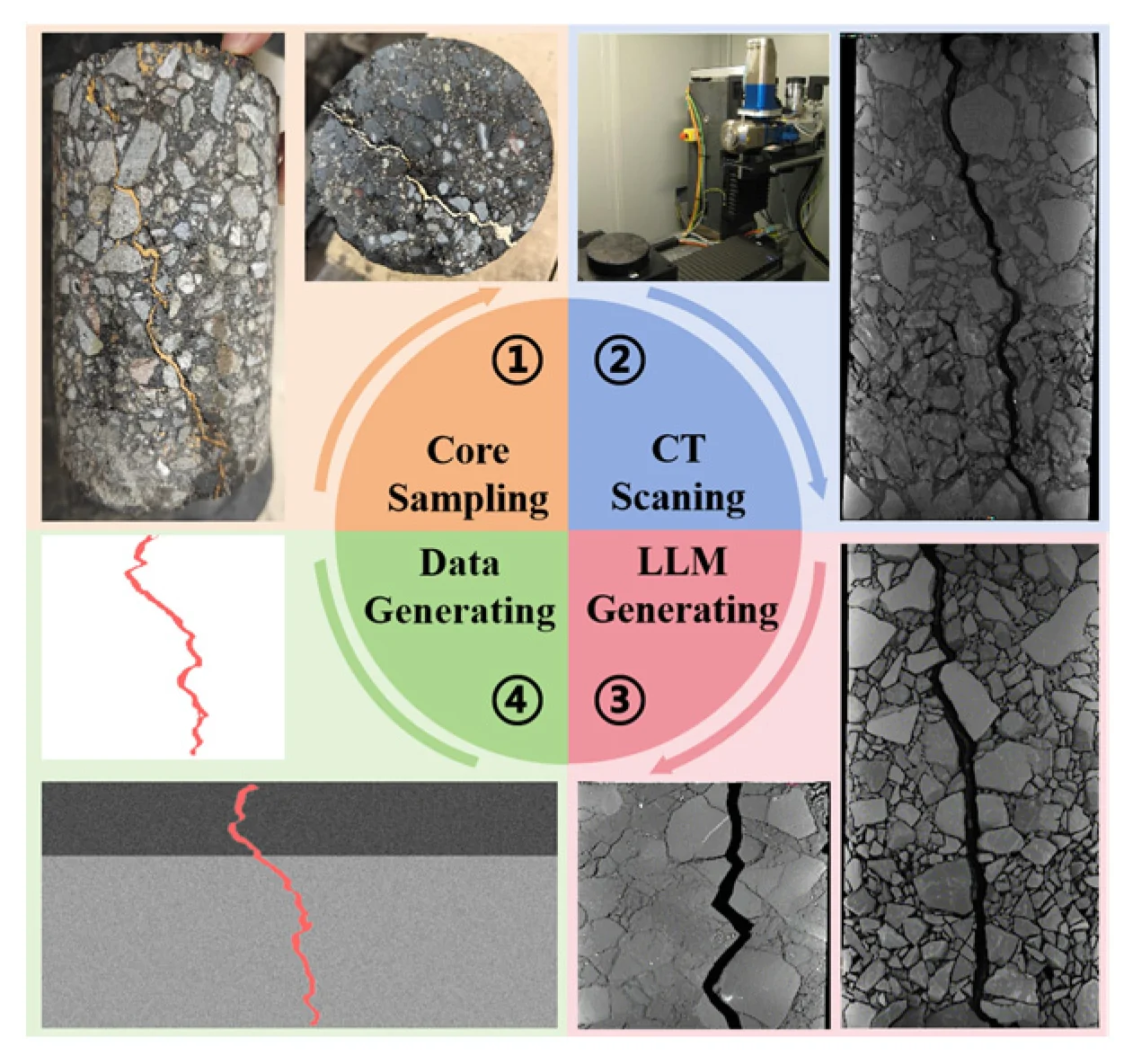
Processing of Crack Morphology Dataset Acquirement.

After CT scanning, the Segment Anything model was employed to segment the crack morphology from the CT images. In total, 60 sets of real crack morphologies from the 10 survey sections were extracted from the core samples. To augment the dataset and enrich the diversity of crack types, an additional 400 synthetic crack images were generated using a generative AI by emulating the structural patterns and extension characteristics observed in real cracks. Simulated background layers were constructed based on the structural features of surface and base pavement layers, and the morphology of cracks was embedded into these backgrounds to form the final set of realistic crack morphology images. The entire process is illustrated in Figure 10. The GPR-CMIGAN dataset consists of two independently constructed and unpaired domains: a GPR domain containing 460 B-scan images and a morphology domain containing 460 crack-morphology images, as shown in Table 1. The equal domain sizes were adopted for balanced training and do not indicate one-to-one correspondence between individual B-scans and morphology images which are divided into training and testing sets at a ratio of 9:1.

**Figure 11:**
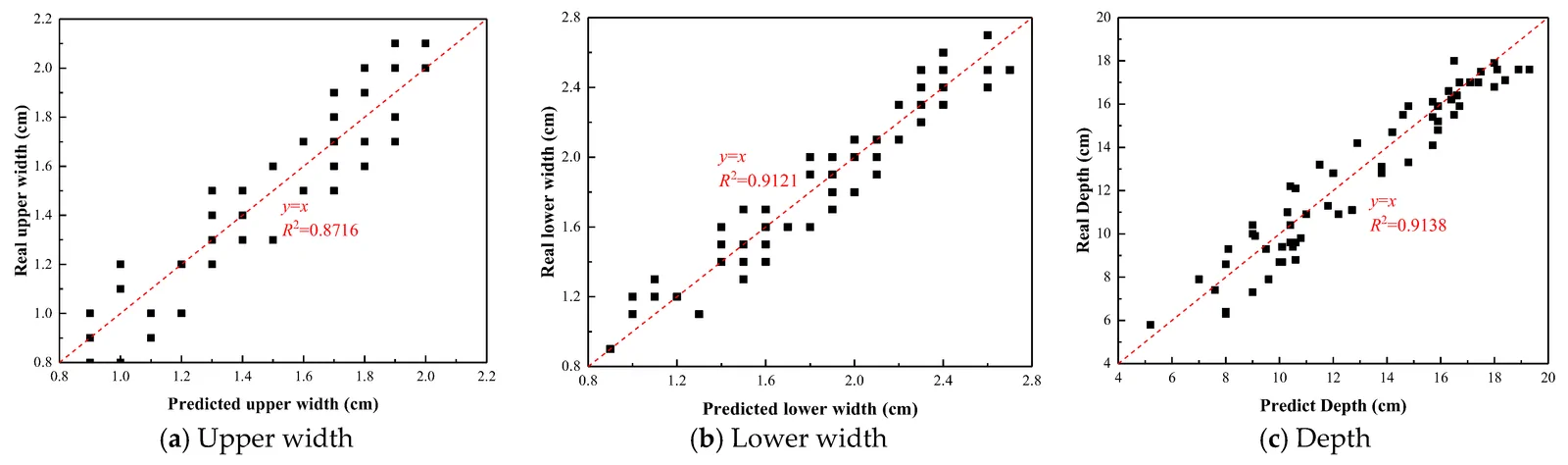
Comparison with calculated crack dimensions and CT-based ground truth.

The CT images of the 60 cores are also used to validate the accuracy of the ground-truth labels using Equations (22)–(29) and Equations (34)–(37). Figure 11 presents the gaps between calculated crack dimensions and CT-based ground truths, including upper and lower crack widths and crack depths. The R^2^ results indicate that ground-truth labels are close to the real crack dimensions.

### 3.3. Training Setting and Evaluation Metrics

#### 3.3.1. Training Setting and Metrics of GPR-GCDNet

The experiments were conducted on a computer running Ubuntu 22.04, equipped with an Intel i5-12600H CPU, 32 GB of RAM, and an NVIDIA RTX 4060Ti-16GB GPU. For GPR-GCDNet, the SGD optimizer was selected, with a learning rate set to 0.01 and a batch size of 16. The GPR-GCDNet was trained for a maximum of 500 epochs when the validation loss did not improve for 5 consecutive epochs. The testing set was evaluated only once after the network architecture and hyperparameters had been finalized.

To evaluate the detection performance of the network, three metrics were adopted: AP, F1 score, and mIoU, with the IoU threshold set to 0.5. AP is used to assess the detection accuracy of the model for target instances. The F1 score is primarily used to evaluate the network’s miss detection and false detection rates. mIoU represents the average IoU between the predicted and labels across all detected instances on the test set. The formulations for these three metrics are:

(38) $$Precision=TP/\left(TP+FP\right)$$

(39) $$Recall=TP/\left(TP+FN\right)$$

(40) $$F1\;Score=2\times Precision\times Recall/\left(Recall+Precision\right)$$

(41) $$AP=\int_{0}^{1}Precision\left(Recall\right)d\;Recall$$

where TP, FP, and FN are the predictions of true positive, false positive, and false negative.

In addition, to assess the computational performance of the model, two metrics were introduced: FLOPs and FPS. FLOPs quantify the number of floating-point computations required to process a single sample, reflecting the computational complexity of the model. FPS denotes the number of samples the model can process per second, serving as an indicator of inference speed. For model size, the count of parameters was used as the evaluation metric, indicating the total number of trainable parameters within the network.

#### 3.3.2. Training Setting and Metrics of GPR-CMIGAN

GPR-CMIGAN is run on the same platform as GPR-GCDNET. The Adam optimizer is employed, with the learning rates for both the generator and discriminator set to 0.01, and the batch size configured as 8. To evaluate the quality of the generated images, five metrics are utilized: MS-SSIM, LPIPS, FID, NRMSE, and PSNR.

As GPR-CMIGAN operates under an unsupervised framework with no paired dataset, it is essential to assess the structural similarity between generated images and real images. MS-SSIM measures pixel consistency across multiple scales by analyzing image means and variances. LPIPS and FID assess perceptual similarity in the feature space using pre-trained neural networks. LPIPS relies on the feature outputs of a specific neural network to evaluate local structural differences. FID computes the distance between feature distributions extracted from intermediate layers of a neural network.

(42) $$MSSSIM\left(\hat{P},P\right)=\frac{1}{m}\sum_{j=1}^{m}\frac{\left(2\mu_{x}^{j}\mu_{y}^{j}+C_{1}\right)\left(2\sigma_{xy}^{j}+C_{2}\right)}{\left({\mu_{x}^{j}}^{2}+{\mu_{y}^{j}}^{2}+C_{1}\right)\left({\sigma_{x}^{j}}^{2}+{\sigma_{y}^{j}}^{2}+C_{2}\right)}$$

(43) $$LPIPS\left(\hat{P},P\right)=\sum_{i}\frac{1}{H_{l}W_{l}}\sum_{h=1}^{H_{l}}\sum_{w=1}^{W_{l}}{\left\|\omega_{l}⨀\left({f_{l}\left(\hat{P}\right)}_{hw}-{f_{l}\left(P\right)}_{hw}\right)\right\|}_{2}^{2}$$

(44) $$FID={\left\|\mu_{x}-\mu_{y}\right\|}^{2}+Tr\left(\sum_{x}+\sum_{y}-2{\left(\sum_{x}\sum_{y}\right)}^{0.5}\right)$$

NRMSE is employed to evaluate the cycle consistency of GPR-CMIGAN, measuring the discrepancy between the input image and the image obtained after sequential processing through both generators. PSNR is used to assess the noise level in the generated images and serves as a direct indicator of image generation quality.

(45) $$NRMSE=\frac{\sqrt{\frac{1}{N}\sum_{i=1}^{N}{\left(\hat{P}-P\right)}^{2}}}{range\;of\;P}$$

(46) $$PSNR=10\times {log}_{10}\left(\frac{255^{2}}{MSE}\right)$$

## 4. Results

### 4.1. Result of GPR-GCDNet

#### 4.1.1. Ablation of GPR-GCDNet

**(1) Hyperparameter Selection of Backbone**

**Figure 12:**
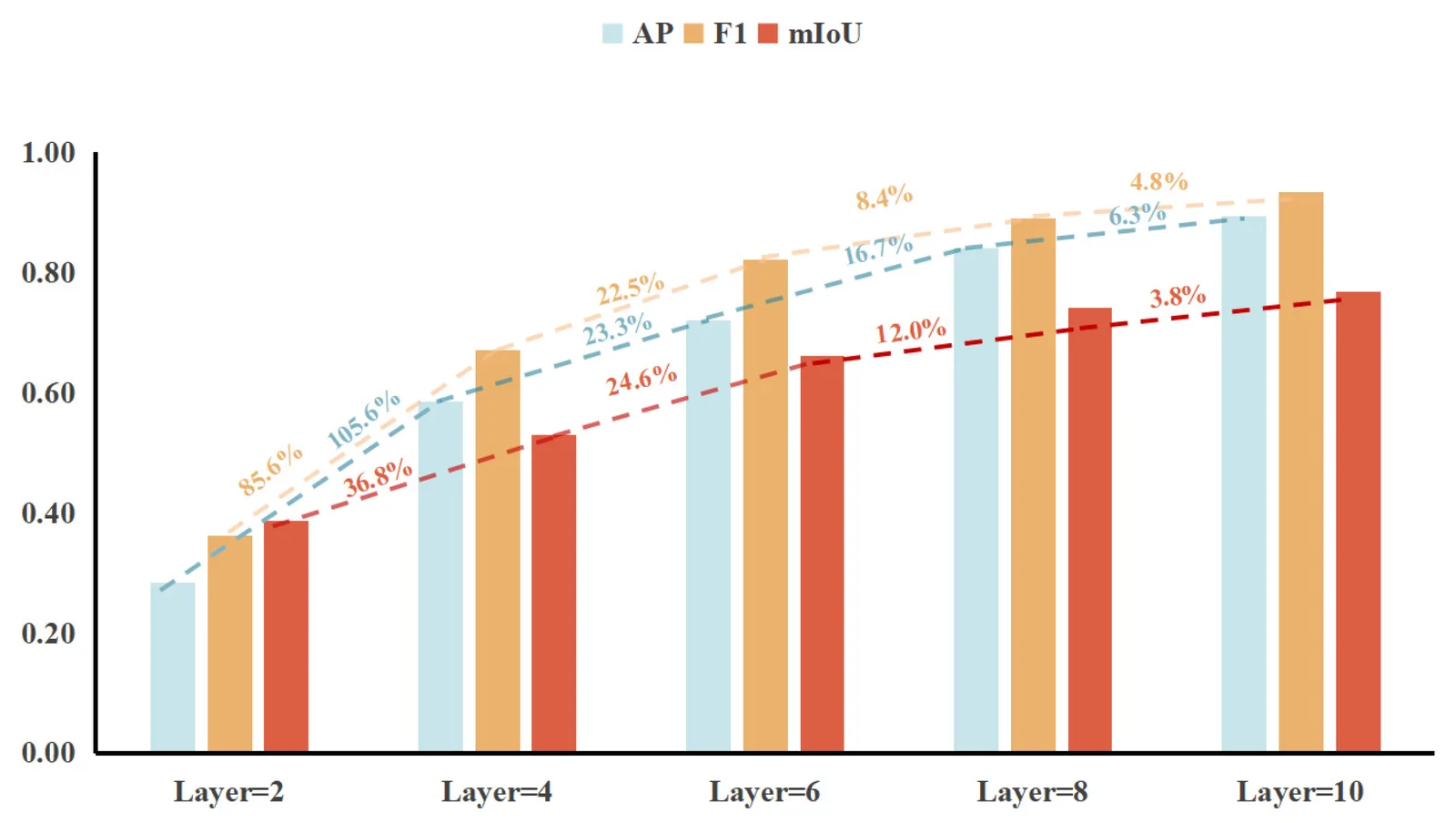
Metrics Comparison of Hyperparameter Ablation Experiment.

In the backbone, the number of layers in Base block#2 has a significant impact on detection performance. A hyperparameter ablation experiment was conducted in this research, wherein different Base block#2 layers were evaluated while keeping all other hyperparameters fixed. Based on the inverted bottleneck structure adopted in the backbone, only even numbers of Base block #2 layers were considered. Consequently, the number of layers was set to 2, 4, 6, 8, and 10. The results of the ablation are presented in Table 2. As the number of layers increases, both AP and F1 score exhibit an improvement. However, the rate of increase diminishes progressively. As shown in Figure 12, when the layer increases from 2 to 4, the AP and F1 scores increase by 105.6% and 85.6%, respectively. In contrast, when the layer increases from 8 to 10, the respective gains reduce to 6.3%and 4.8%, revealing a “marginal effect” between depth and accuracy.

At the same time, increasing the number of Base block#2 leads to an increase in computational cost. When the layer increases from 2 to 4, the FLOPs increases by only 0.54 G. However, when the layer increases from 8 to 10, the computational cost increases by 4.75 G, primarily due to the channel doubling within each added Base block#2. Considering both detection performance and computational efficiency, it was observed that setting layer = 8, a 16.7% increase in accuracy at the cost of a 6.7% increase in FLOPs. When setting layer = 10 provides a 6.3% AP increase with a 10.4% increase in FLOPs. Therefore, layer = 8 was selected as the optimal configuration for feature extraction.

**(2) Multi-scale Feature Fusion Ablation**

**Figure 13:**
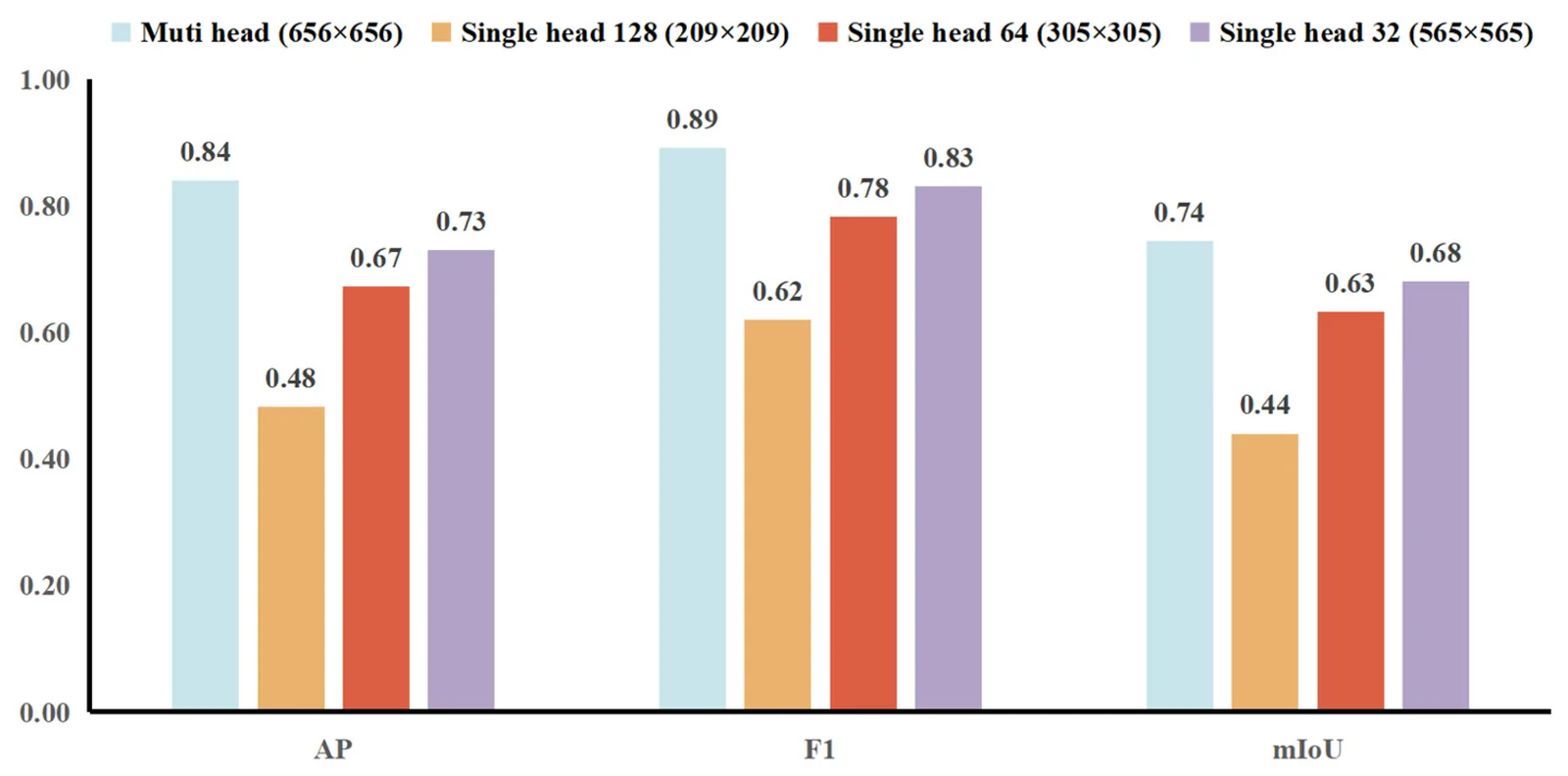
Comparison of evaluation metrics in the multi-scale feature-fusion ablation study.

An ablation was designed in this study to evaluate the impact of the multi-scale feature fusion structure within the detection head branch on detection performance, as shown in Figure 13. The backbone network includes three feature branches of different spatial resolutions (128 × 128, 64 × 64, and 32 × 32), which are subsequently fused through the Neck module to enhance multi-scale representation. In the ablation experiment, each feature branch was individually isolated and directly passed to the SetTrap head for detection. As shown in Figure 12, using single-scale input significantly degraded detection accuracy. The 128 × 128 branch yielded an AP of only 0.48, which corresponds to 57.1% of the performance achieved with the multi-scale feature fusion. The 64 × 64 and 32 × 32 branches achieved AP values of 0.67 and 0.73, respectively. The reduction in mIoU further demonstrates that single-scale features are insufficient for comprehensive recognition of structural cracks. The F1 score exhibited a similar downward trend to AP, indicating a substantial increase in both miss detection rate and false detection rate. These results confirm the significant advantages of the multi-scale fusion structure in terms of both detection accuracy and recall performance.

**Figure 14:**
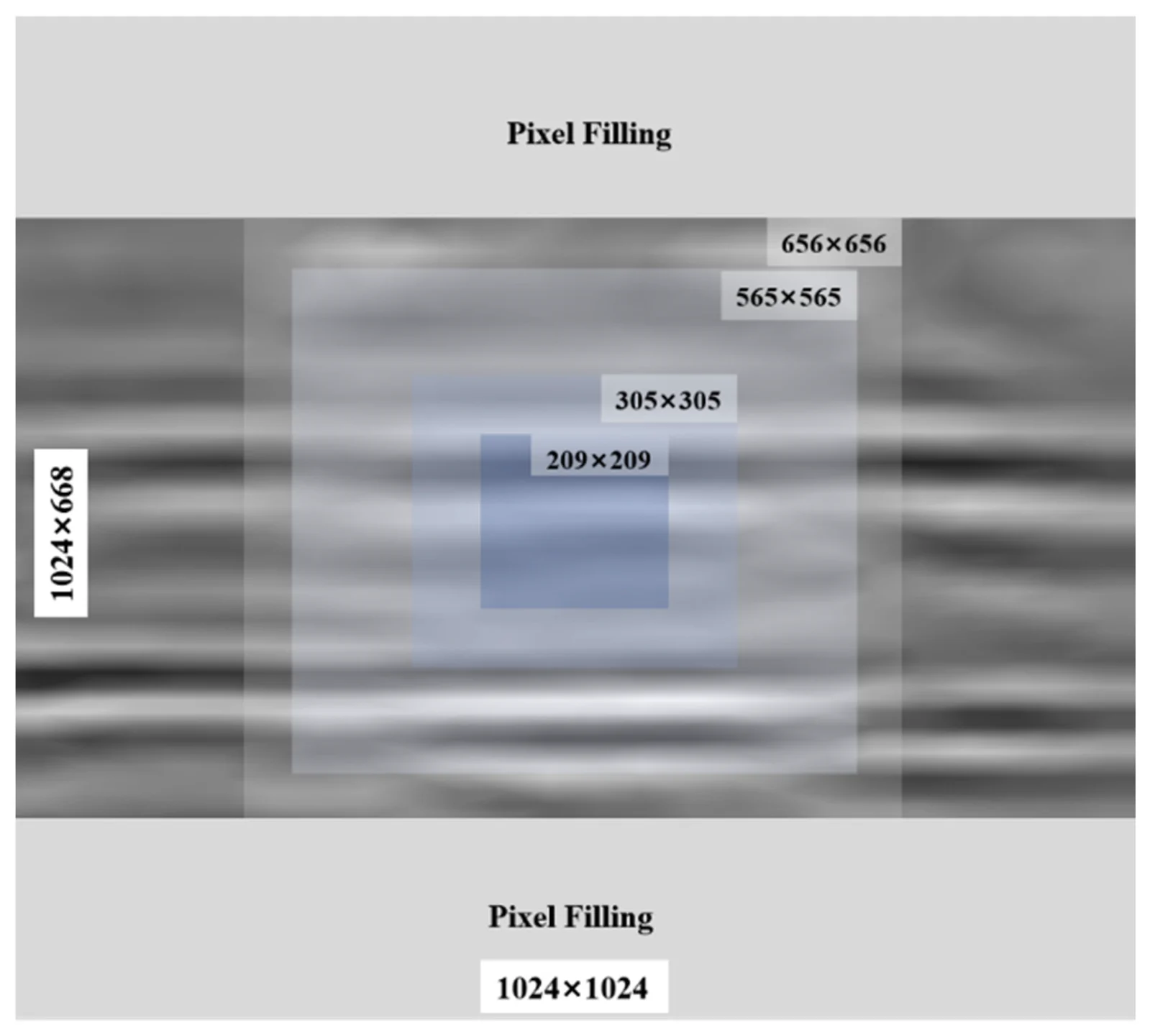
Comparison of receptive fields for single-scale and multi-scale feature representations.

Performance improvement primarily stems from the enhanced deep feature representations provided by multi-scale feature fusion, as shown in Figure 14. The Neck module facilitates feature sharing across different spatial resolutions, thereby strengthening both local and global feature expressiveness, which in turn enables more effective recognition of hyperbolic signals. In addition, the enhancement in detection performance is closely associated with the receptive field size. The receptive field of GPR-GCDNet has been estimated to be approximately 656 × 656. As illustrated in Figure 13, this receptive field is enough to cover the entire region of the structural crack’s hyperbolic patterns. In comparison, the receptive fields of the three individual branches measure 209 × 209, 305 × 305, and 565 × 565, respectively. Detection performance improves with increased receptive field size, further validating that a larger receptive field enables the network to search for target features over a broader spatial context. On one hand, in B-scan, the hyperbolic patterns typically occupy a considerable area, and a larger receptive field contributes to capturing their complete structural patterns. On the other hand, the task involves geometrical parameter estimation, which places a stronger dependence on global contextual information. In summary, the multi-scale feature fusion is critically important for enhancing detection accuracy in the context of this task.

**(3) Directional Crack Attention Ablation**

**Figure 15:**
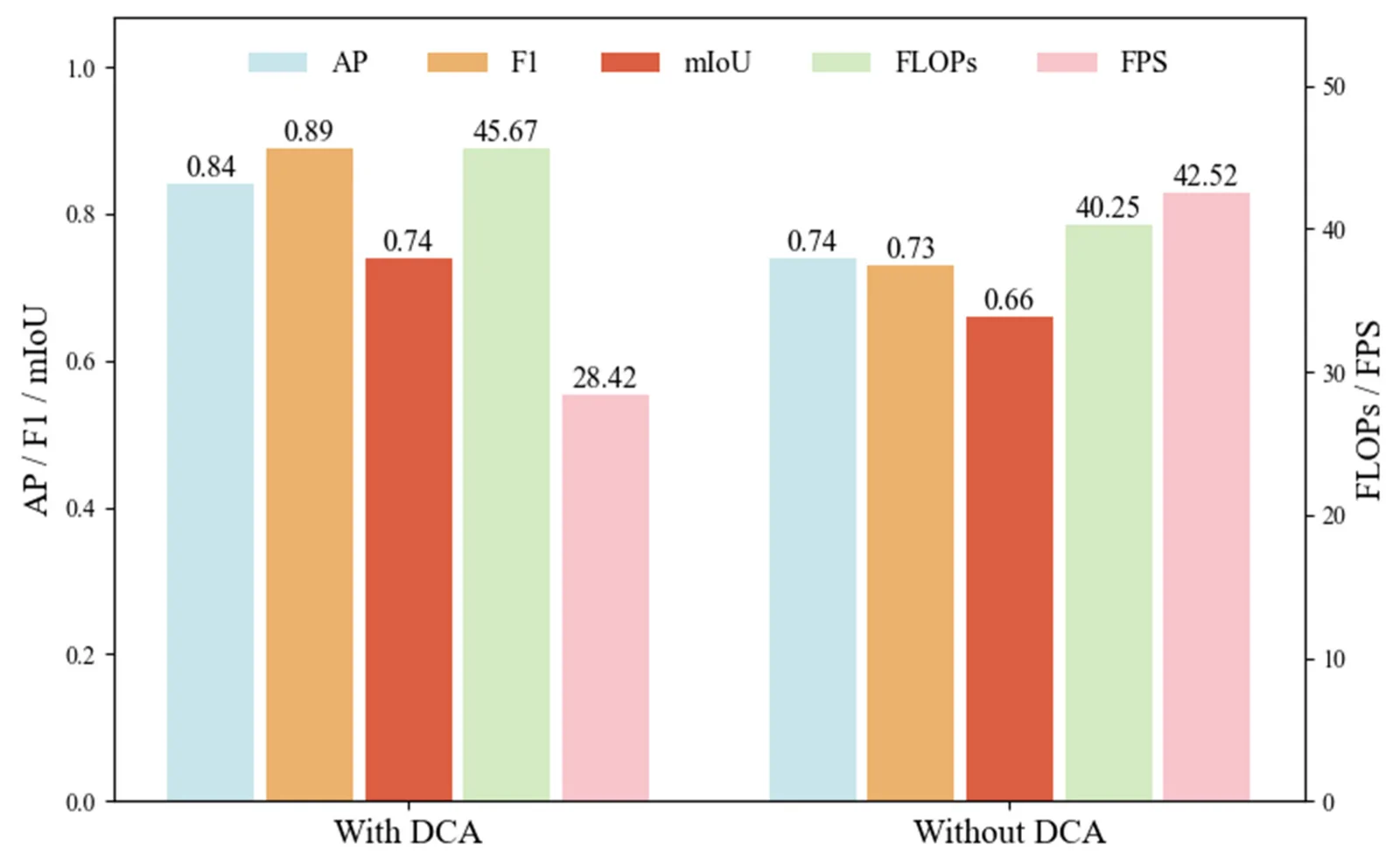
Metrics Comparison of DCA Ablation Experiment.

To evaluate the impact of DCA, an ablation experiment was conducted in which all DCA modules were removed from GPR-GCDNet. The results, presented in Figure 15, indicate that DCA plays a critical role in enhancing detection accuracy. After the removal of DCA, the AP decreased from 0.84 to 0.74, representing a 11.9% reduction. Similarly, the F1 score decreased from 0.89 to 0.73, corresponding to a 18.0% reduction. These findings suggest that DCA not only improves the overall detection capability of the model but balances between precision and recall. The reduction in F1 score means high miss detection and false detection, indicating degraded robustness in the model’s detection performance.

**Figure 16:**
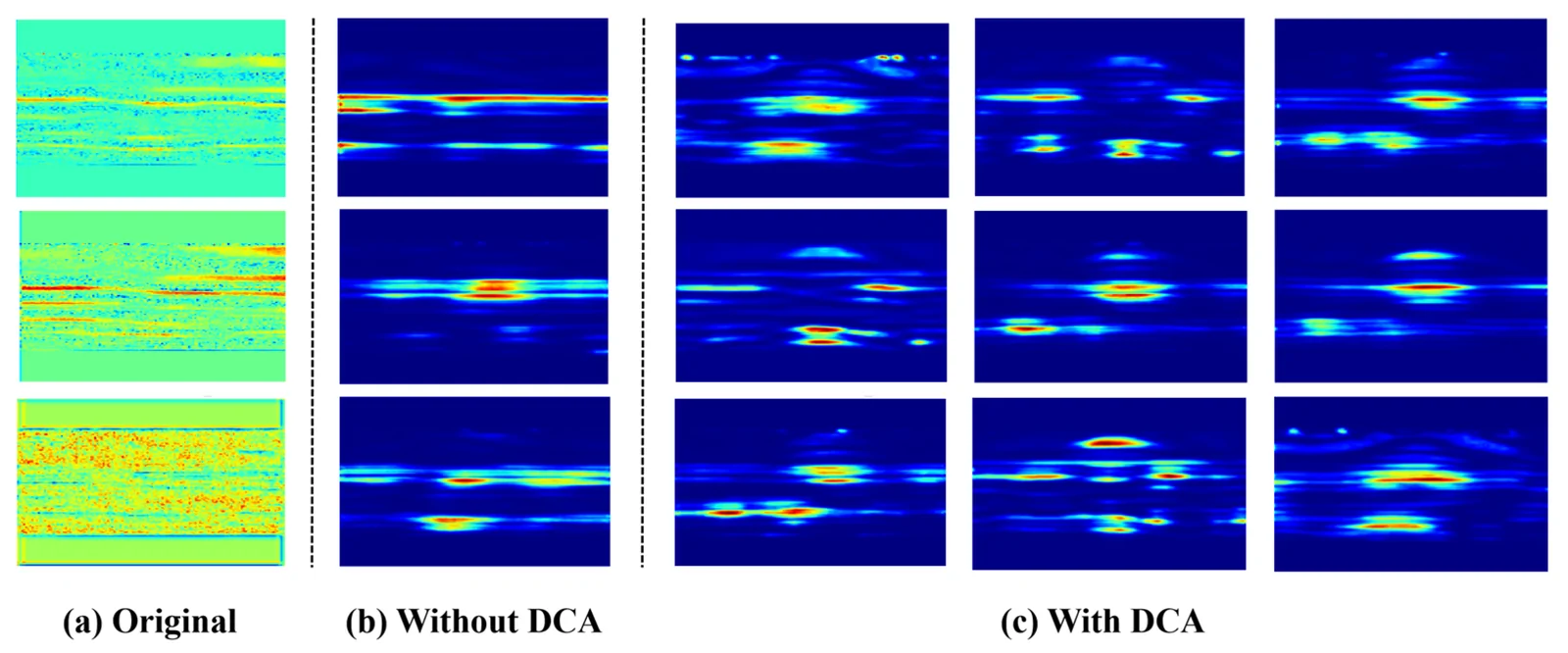
DCA ablation: attention heatmaps with and without DCA.

The trapezoidal boxes occupy only 3% to 5% of the image area, indicating that the target regions are extremely sparse within the overall feature map. As a result, uniform attention is insufficient for focusing on these critical regions. Moreover, the trapezoidal boxes exhibit scale asymmetry along the *x*-axis and *y*-axis. Based on the morphological characteristics of cracks, the *x*-axis scale of a crack in a B-scan is significantly smaller than its *y*-axis, making the *x*-axis localization particularly susceptible to tiny disturbance, which can cause substantial deviation in positioning accuracy. The DCA addresses this challenge by assigning spatial attention weights across the global receptive field, thereby enhancing the network’s responsiveness to hyperbolic regions. Furthermore, DCA independently applies *x*-axis weights, enabling the model to extract lateral features, thus improving localization precision along the *x*-axis. Figure 16 presents the attention heatmaps generated by the model. Subfigure (a) shows the initial random attention, while subfigure (c) demonstrates the focused attention output when DCA is applied, clearly highlighting the hyperbolic pattern with stronger emphasis. In contrast, (b) shows that although the model without DCA can correctly identify the structural layer, the *x*-axis attention remains diffuse, and *y*-axis attention is sparse. DCA significantly improves the accuracy and stability of crack dimension detection.

However, the incorporation of DCA not only adds additional convolutional layers to generate adaptive directional weights but also performs element-wise matrix operations on the output feature maps, which significantly reduces the computational efficiency of the model. As a result, the computational cost increases from 40.25 G to 45.67 G. Moreover, the inference speed decreases from 42.52 FPS to 28.42 FPS, representing a 33.2% reduction.

#### 4.1.2. Comparison Study with Advanced Detection Approaches

**Figure 17:**
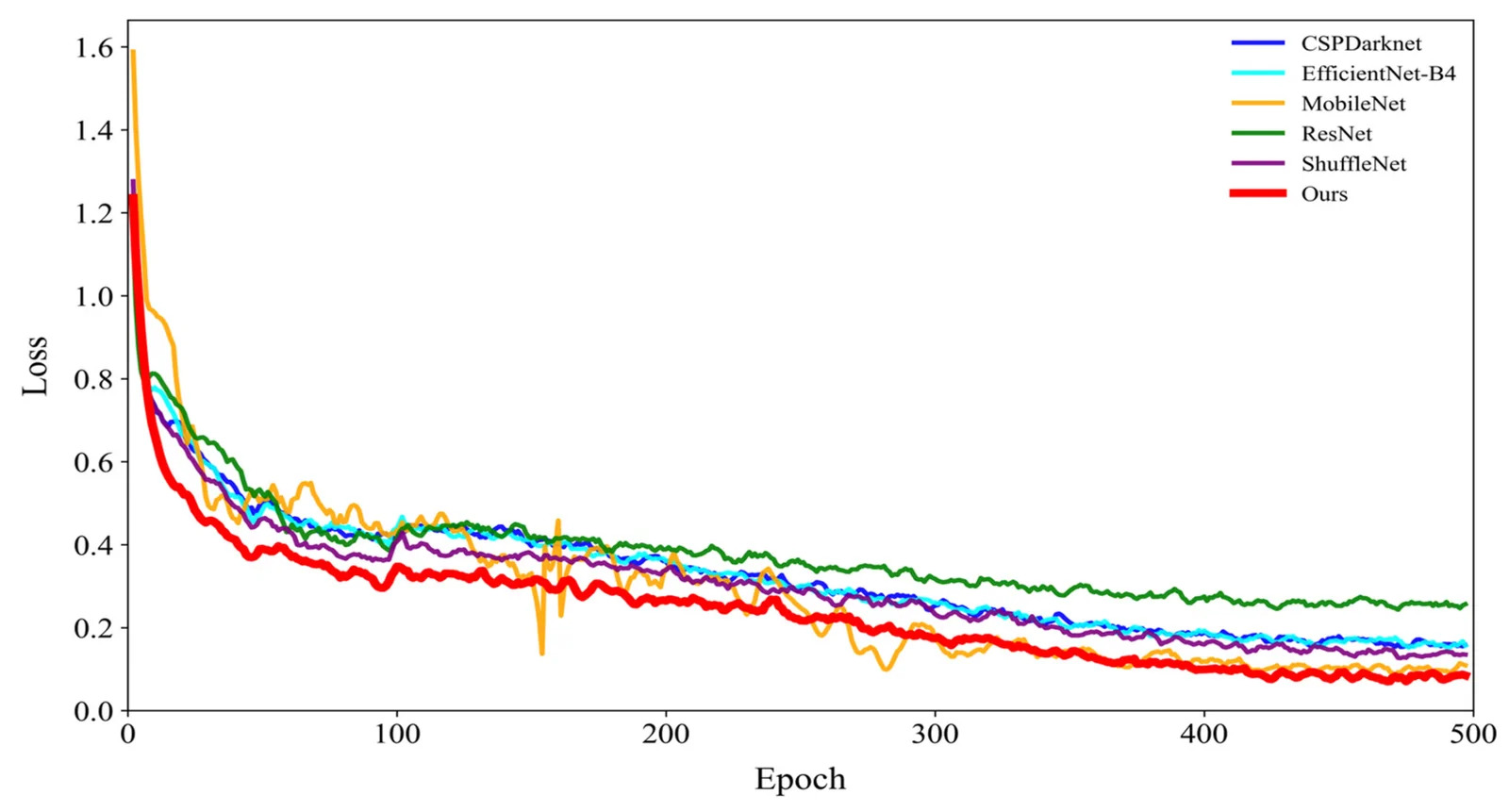
Training loss curves of GPR-GCDNet and the comparison models.

To systematically evaluate the performance of the proposed GPR-GCDNet, a series of comparison experiments were designed and conducted. Five representative backbone network architectures were selected to replace the original backbone and neck modules in GPR-GCDNet, to explore the applicability and effectiveness of different feature extraction strategies for this task. The selected backbone networks include CSPDarknet [35], ResNet101 [36], MobileNet-v4 [44], EfficientNet-B4 [45], and ShuffleNet [46]. All models were trained under identical datasets and training configurations to ensure fair comparison. Figure 17 illustrates the loss curves during training for each network, providing insight into their respective convergence speeds and training stability.

Table 3 presents the performance of each network under three metrics: AP, F1 score, and mIoU. The results indicate that GPR-GCDNet achieves an AP of 0.84 at an IoU threshold of 0.5, outperforming all baseline networks and demonstrating a clear advantage in detection accuracy. Among the comparative models, CSPDarknet, which also incorporates a multi-scale feature fusion strategy, shows relatively strong performance, further confirming the importance of multi-scale information in the detection of crack geometrical dimensions. In contrast, architectures such as ResNet101, MobileNet-v4, and ShuffleNet, though well-established in general visual tasks, exhibit lower AP and F1 scores in this context, primarily due to limitations in feature extraction depth and receptive field coverage. The F1 score trends are similar to AP, suggesting it achieves a better balance between miss detection and false detection rates. At an IoU threshold of 0.5, GPR-GCDNet achieves an F1 score of 0.89, surpassing all other backbone networks and further validating its robustness in real-world crack detection scenarios. Moreover, GPR-GCDNet attains a mIoU of 0.74 across all correctly detected samples, thereby reinforcing its performance advantage in crack dimension detection tasks.

Figure 17 illustrates the detection performance of each model on representative samples. GPR-GCDNet demonstrates superior performance in terms of localization accuracy and dimension detection within crack regions, reflecting its enhanced feature representation and matching capabilities. For instance, in Sample⑥, both ShuffleNet and EfficientNet-B4 exhibit noticeable inaccuracies, MobileNet-V4 and ShuffleNet display evident deviations, while ResNet-101 retains multiple trapezoidal boxes, indicating over-detection. In contrast, GPR-GCDNet consistently delivers more precise and reliable detections across all evaluation aspects.

**Figure 18:**
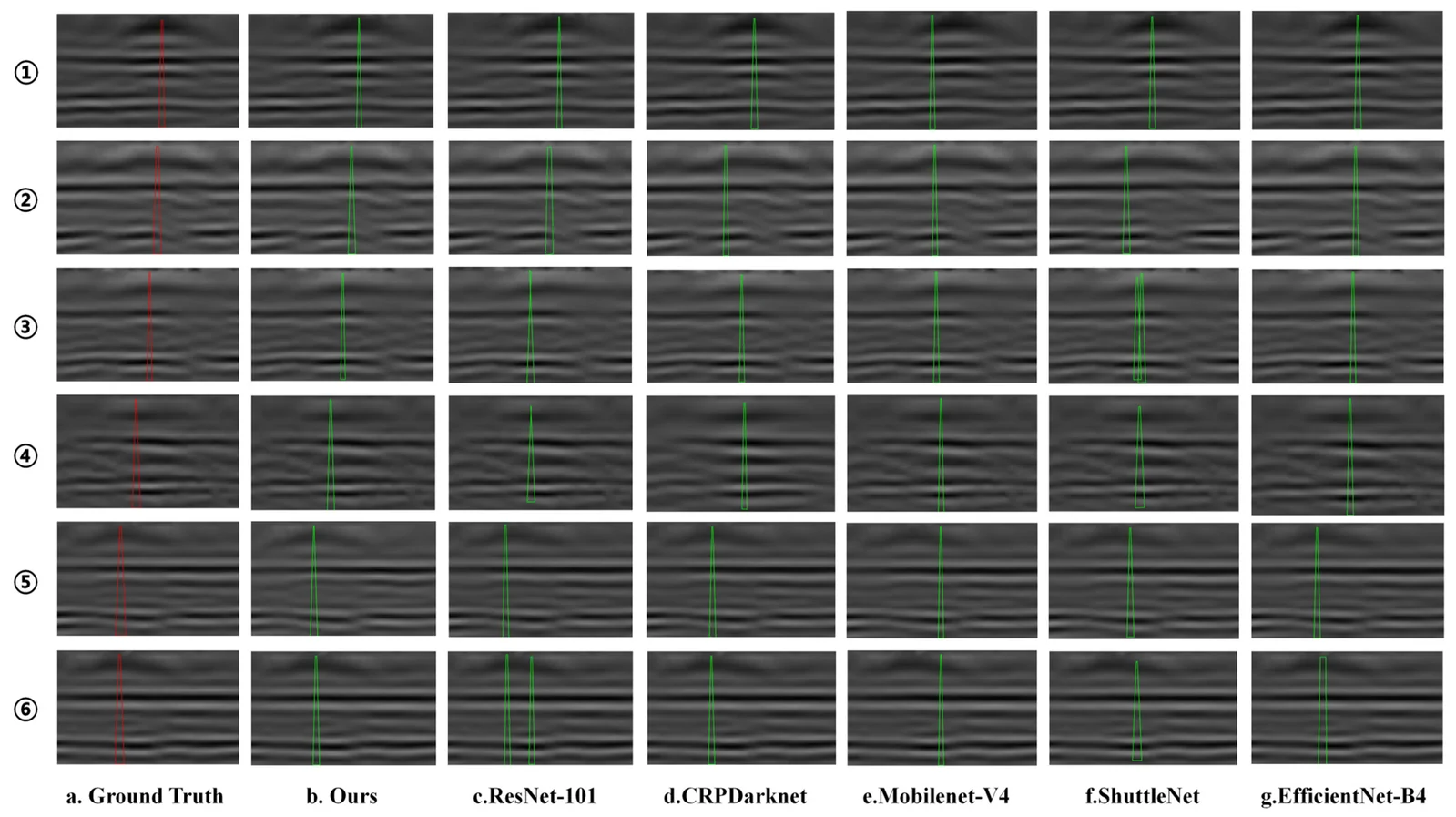
Demonstration of Model Detection Samples, where the red and green boxes are the ground truths and detection results.

As observed in Figure 18, the trapezoidal boxes differ fundamentally from conventional bounding boxes. First, they fail to fully encompass the hyperbolic reflection regions typically exhibited by structural cracks in B-scan. Second, due to complex morphology, and indistinct boundaries of crack regions within B-scan, the detection task imposes significantly greater demands on the model’s ability to capture spatial information. Using local features only proves insufficient in such cases, as a limited receptive field often leads to missed detections or inaccurate dimension detections. In Section 4.1.2, the critical role of a large receptive field was analyzed. Furthermore, Section 4.1.3 examined the effect of DCA in improving spatial attention allocation. In addition to these factors, the design of the SetTrap head plays an important role. Unlike traditional object detection approaches that rely on a grid-based anchor box, GPR-GCDNet adopts a Set Prediction strategy, wherein many candidate boxes are generated directly during inference. The Hungarian matching algorithm is then employed to perform the global optimal assignment of positive and negative samples among these candidates. This approach not only simplifies post-processing but also allows the model to flexibly learn a bounding strategy better aligned with the irregular morphology of cracks, thereby broadening the detection scope and enhancing recall performance. In summary, the integration of a large receptive field and a SetTrap head significantly enhances the model’s capacity to perceive complex crack regions, resulting in improvements in both detection accuracy and robustness.

However, the detection efficiency of GPR-GCDNet is lower compared to other models. The model comprises 66.51 million parameters, a quantity comparable to other backbone architectures; however, its computational cost reaches 48.32 G, indicating a high resource demand. This issue primarily arises from the introduction of numerous high-channel feature maps in the intermediate layers. Experimental results show that the average inference speed of GPR-GCDNet is 28.42 FPS. While this is enough to meet the real-time requirements of 24-channel GPR systems, there remains room for improvement. Therefore, how to further optimize the computational cost, and reduce redundant feature channels remains a critical direction for future research.

**Figure 19:**
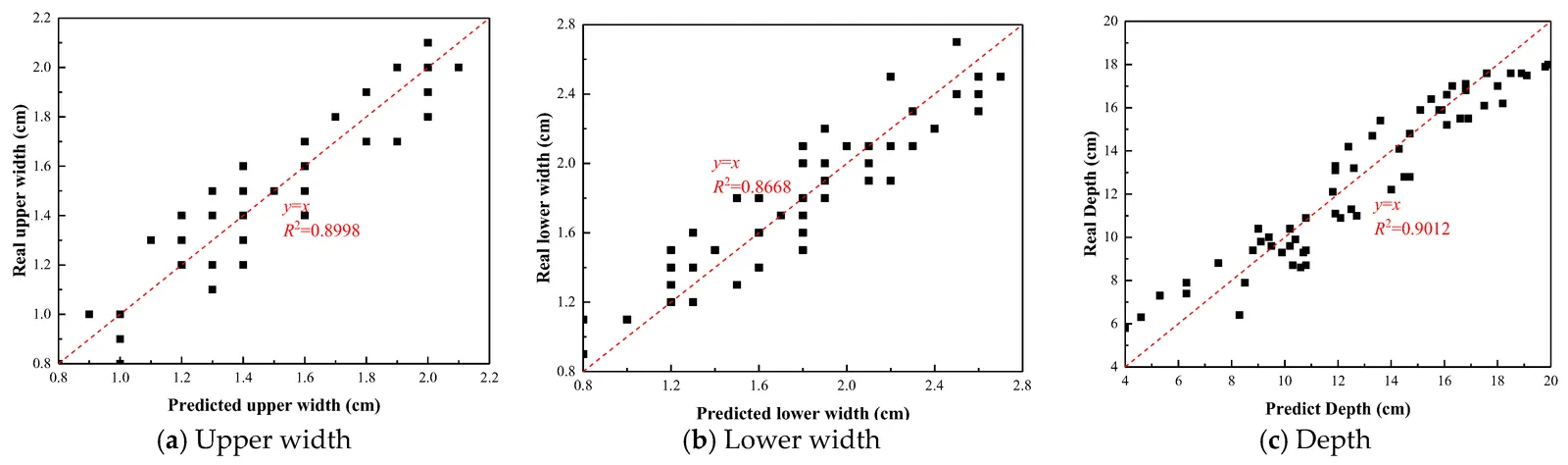
Comparison with predicted crack dimensions and CT-based ground truths.

Finally, the predicted crack dimensions value is compared with the CT-based ground truths, including upper and lower crack widths and crack depths, as shown in Figure 19. Figure 19 compares the crack dimensions predicted by GPR-GCDNet with the CT-derived measurements for the independently held-out core specimens. All CT images, corresponding B-scans, cropped samples, and augmented samples from these cores were excluded from model training and hyperparameter selection. The results show agreement between the predicted and CT-derived upper opening widths, lower opening widths, and crack depths. Nevertheless, the differences between Figure 11 and Figure 19 reflect the combined effects of uncertainty in the analytically calculated labels and prediction error introduced by GPR-GCDNet. These two sources of error cannot be separated solely by comparing their R^2^ values. The R^2^ results in Figure 19 indicate that the predictions are close to the real crack dimensions This demonstrates that the ground-truth labels can still be improved, though they are close to the real dimensions.

The CT-based comparisons in Figure 11 and Figure 19 directly quantify the accuracy of the estimated crack dimensions in physical units. Figure 11 compares the equation-derived geometrical labels with the CT-derived measurements, whereas Figure 19 compares the GPR-GCDNet predictions with the same types of physical measurements. In general, the GPR-GCDNet achieves MREs of 9.67%, 11.23%, and 9.12% and R^2^s of 0.8998, 0.8668, and 0.9012 in the terms of upper opening width, lower opening width, and crack depth, respectively. These results demonstrate the degree of agreement between the GPR-derived dimensions and the geometrical characteristics measured from actual pavement cores.

#### 4.1.3. Stability Analysis

**Figure 20:**
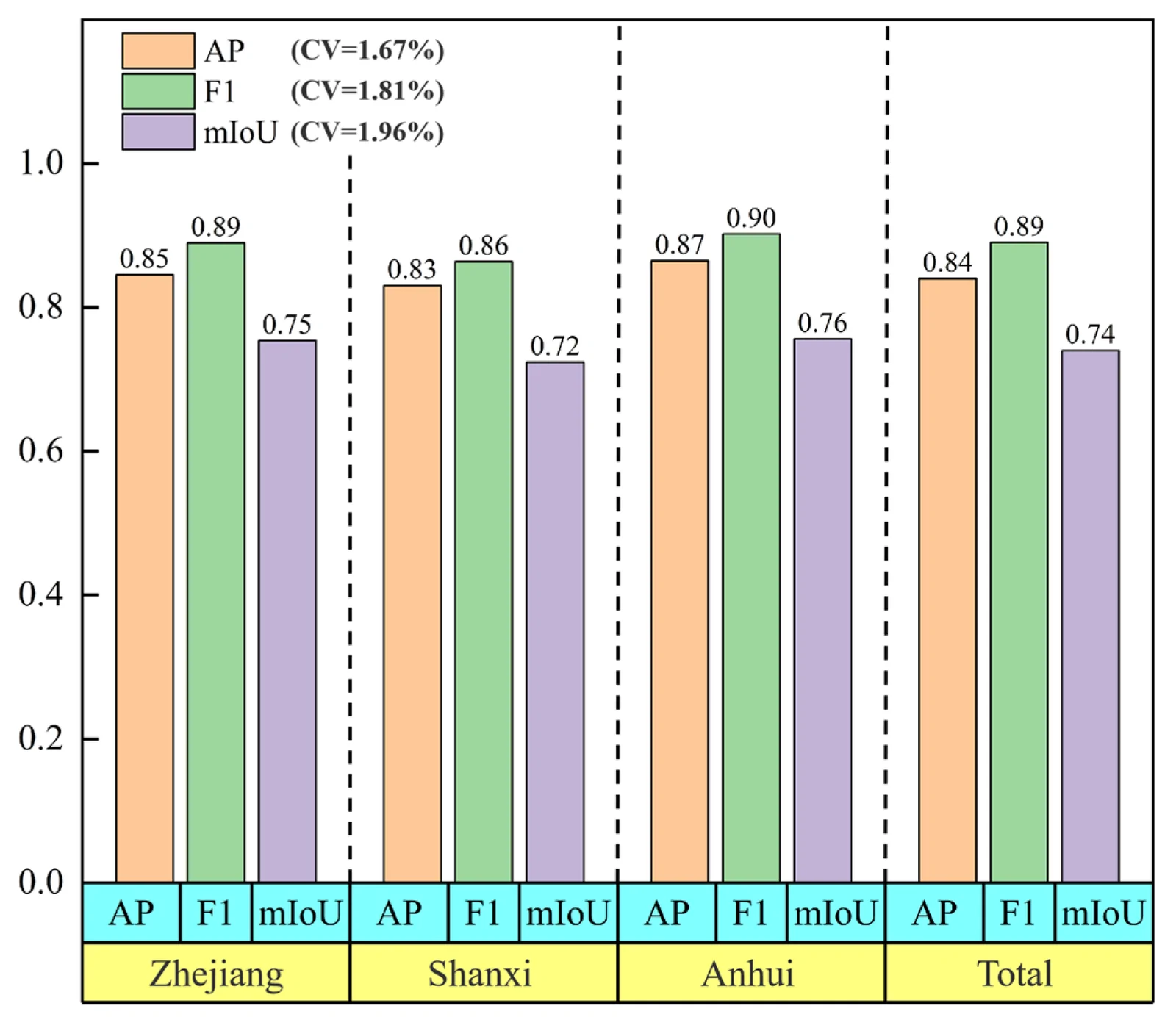
Stability Analysis of GPR-GCDNet.

To evaluate the robustness of GPR-GCDNet under varying pavement structures, it was tested by data from three different highways in Zhejiang, Anhui, and Shanxi provinces. Figure 20 presents the quantitative comparison of detection results across the three test sites in terms of AP, F1 score, and mIoU. Overall, the detection results exhibit a high degree of consistency across different highways. The best performance was observed on the highway in Anhui, where the AP, F1 score, and mIoU reached 0.87, 0.90, and 0.76, respectively. While performance on the highway in Shanxi was slightly lower, the differences remained minimal, with respective reductions of only 4.6%, 4.9%, and 5.3%.

To assess performance fluctuations, the coefficient of variation (CV) was computed for the three metrics across the three datasets. Results show that AP exhibited the lowest CV at 1.67%, indicating the highest stability in detection accuracy. The mIoU recorded a CV of 1.96%, the highest among the three, though still within a low range. Collectively, all three metrics exhibited CV values below 2%, confirming that the model delivers consistent and reliable detection performance across diverse pavement structures.

Since the samples were from 10 different surveys and 3 different province highways, this experiment evaluates performance consistency among the represented survey environments in China. Therefore, the future work should expand the dataset from more pavement structures and environments.

The relatively small dataset compared with the complexity of GPR-GCDNet represents a potential source of overfitting. GPR-GCDNet contains 66.51 million parameters but is trained using 493 original training samples from a total dataset of 704 B-scans. Source-level partitioning, training-only augmentation, dropout, validation-based early stopping, and one-time test evaluation were adopted to reduce this risk. The consistent results across the three provincial datasets provide evidence of generalization under the represented survey conditions. Nevertheless, the test subset contains only 70 samples from 10 survey sections, and the cross-site analysis is not equivalent to validation on a large external dataset. Therefore, the reported generalization should be limited to reflective cracks in pavement structures and survey environments similar to those represented in the current dataset.

### 4.2. Result of GPR-CMIGAN

To comprehensively evaluate the capability of the proposed GPR-CMIGAN in the task of crack morphology inversion, a series of six controlled experiments were systematically designed. These experiments encompass the full progression from the baseline architecture to progressively refined variants. The six models are as follows: (1) CycleGAN; (2) U-Net CycleGAN in which CycleGAN generator is replaced with a U-Net generator; (3) CycleGAN with Gaussian noise injection; (4) U-Net CycleGAN with Gaussian noise injection; (5) U-Net CycleGAN network with crack-dimension-constrained cycle-consistency loss; (6) GPR-CMIGAN integrating Wasserstein Loss and all improvements.

**Figure 21:**
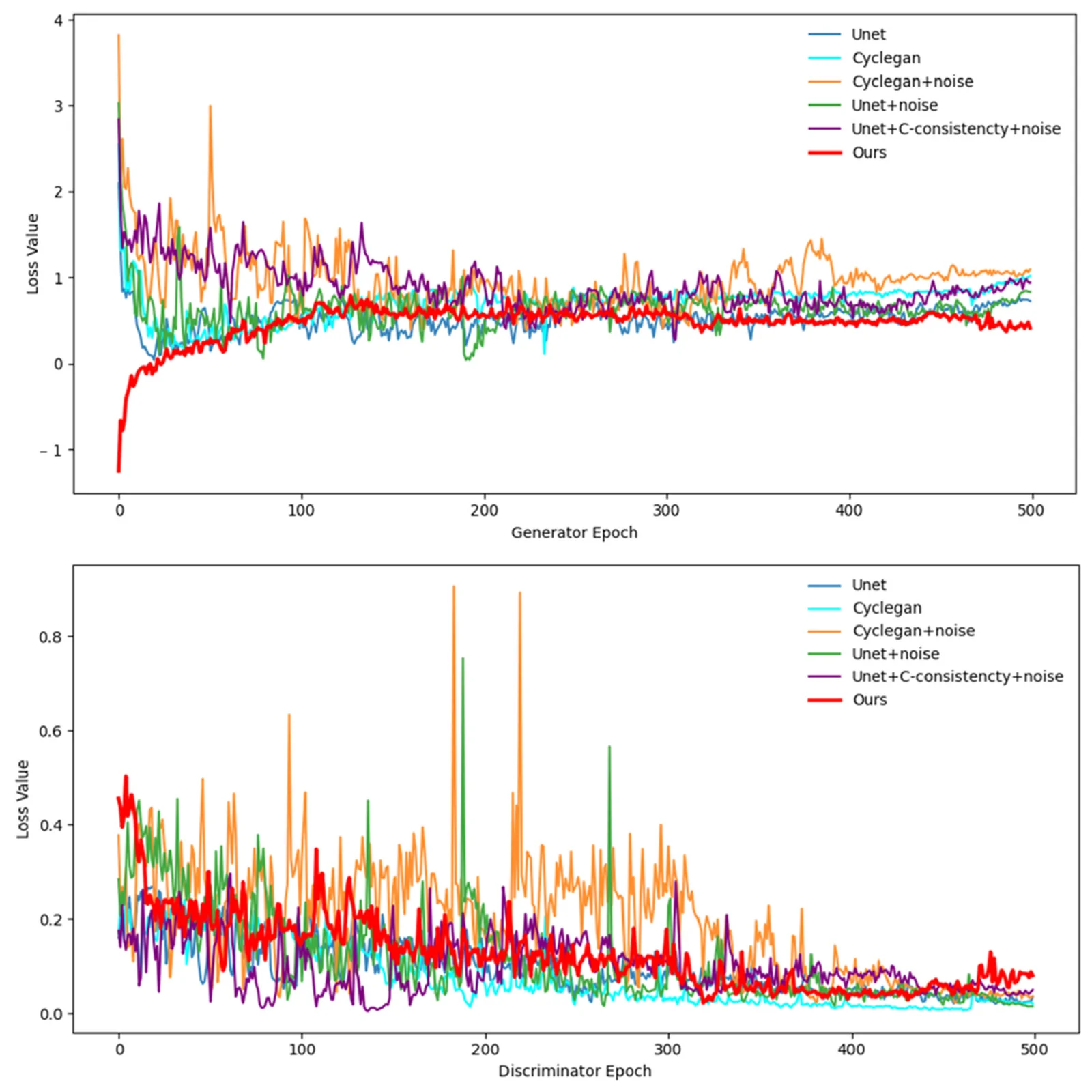
Loss Curve of GPR-CMIGAN.

Figure 21 presents the generator and discriminator loss curves across the different models. For GPR-CMIGAN, due to the utilization of Wasserstein Loss, the adversarial loss dominates the generator’s loss in the early training stages, resulting in initial negative values for the total generator loss. As training progresses, the adversarial loss gradually approaches zero, allowing the positive-valued components to become dominant, ultimately driving the generator loss to positive values, followed by a gradual decline and convergence. Following the principles of generative adversarial learning, the generator and discriminator losses exhibit correlated trends. Table 4 summarizes the quantitative performance of each model on the testing set, while Figure 20 provides the visual comparisons of generated crack images across all model variants.

**Figure 22:**
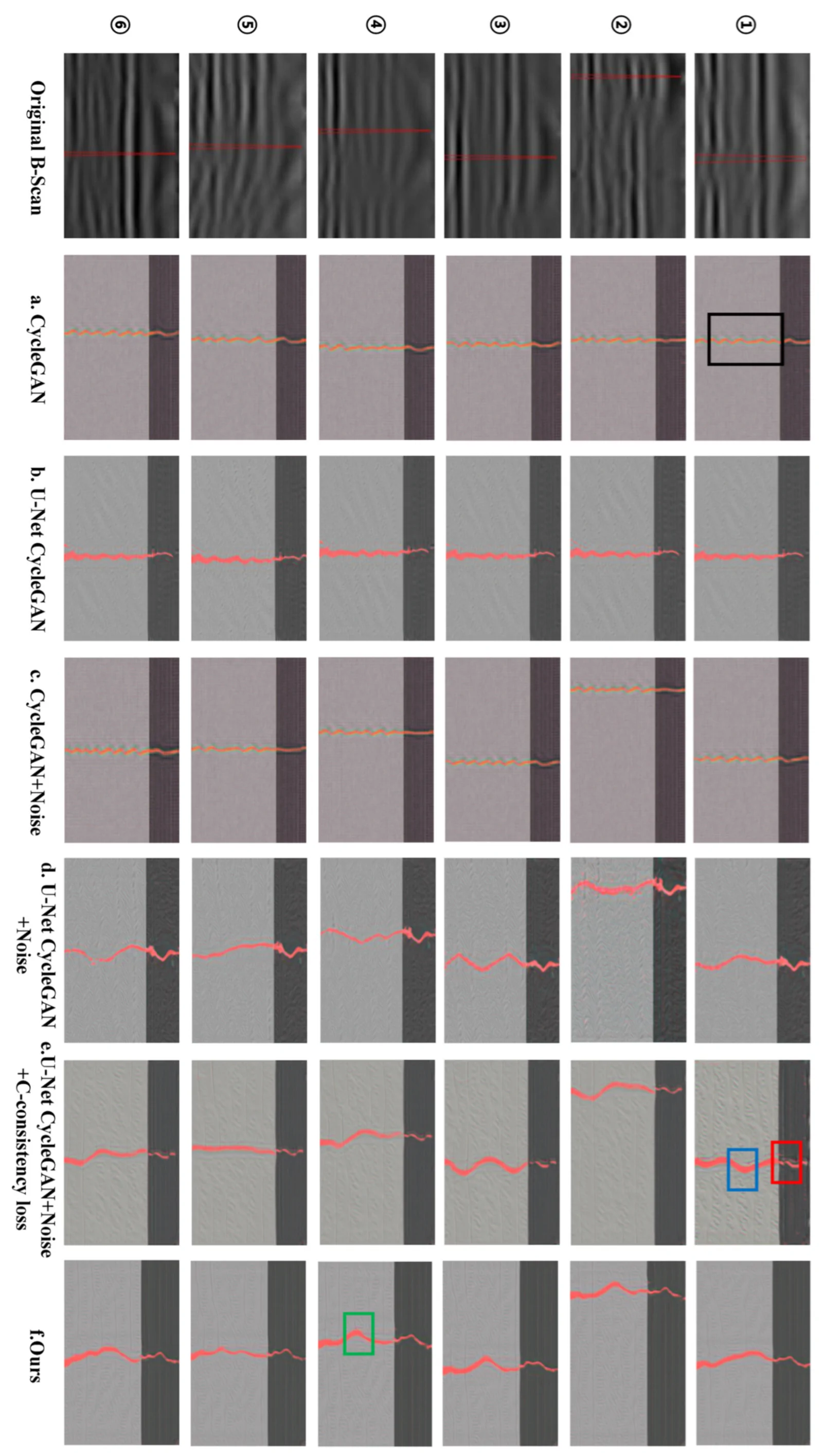
Demonstration of GPR-CMIGAN Generation Samples, where the red regions are the predicted crack areas and the read boxes in the first raw are the labels.

Comparative analysis reveals that replacing the CycleGAN generator with a U-Net generator results in significant improvements across multiple metrics assessing image quality and structural similarity. Notable gains were observed in MS-SSIM, LPIPS, and FID, with improvements of 9%, 20%, and 22%, respectively, compared to the original CycleGAN. These results indicate that the generated images by the U-Net generator exhibit higher pixel-level structure consistency and perceptual consistency with the target image domain. The U-Net generator, by leveraging skip connections, effectively preserves initial features while enhancing semantic representation, contributing to more realistic image generation. The NRMSE of 0.3098 achieved by the U-Net CycleGAN model reflects a high degree of cycle consistency between the two generators. Additionally, the PSNR improved by 14.7047, indicating more effective noise suppression and overall enhancement in image quality. However, as shown in Figure 22, both CycleGAN and U-Net CycleGAN exhibit signs of mode collapse. The generated cracks across five samples appear in nearly identical regions, with highly similar widths and morphology. This phenomenon indicates a deterministic mapping behavior in which models fail to generalize variations in the input distribution.

GPR-CMIGAN is also compared with other advanced inversion methods, as shown in Table 4. GPR-CMIGAN achieved the best MS-SSIM, LPIPS, and FID values. Compared with the strongest baseline, it improves MS-SSIM and PSNR by 7.7% and 1.0295, respectively, while it reduces the LPIPS, FID, and NRMSE by 1.66%, 0.2487, and 1.0295, respectively. These results indicate that the proposed dimensional constraint improves both image quality and the geometrical consistency of the reconstructed crack morphology.

The above results validate GPR-CMIGAN at the image-domain, distribution, cycle-reconstruction, and dimensional-consistency levels. However, they should not be interpreted as a specimen-specific comparison between an inverted morphology and its spatially corresponding CT morphology. Because the B-scan and CT domains used by GPR-CMIGAN are unpaired, the internal branching pattern, tortuosity, local orientation, and propagation path of an individual concealed crack cannot be uniquely verified using the present dataset. Consequently, the generated result represents a dimension-constrained plausible morphology that is consistent with the learned CT crack domain, rather than an exact physical ground truth for the corresponding B-scan. Future work will require spatially registered B-scan, pavement-core, and CT datasets to perform direct pixel-, boundary-, and topology-level validation.

The CT-based comparison in Figure 19 physically validates the dimensional constraints supplied to GPR-CMIGAN but does not constitute a specimen-specific validation of the complete reconstructed morphology. Because the B-scan and CT domains are unpaired, direct validation of local crack shape, branching, connectivity, and internal spatial characteristics requires spatially registered B-scan–core–CT data.

The generalization of GPR-CMIGAN is additionally constrained by the limited scale and composition of its morphology domain. Only 60 of the 460 morphology images were directly derived from CT scans of physical cores, whereas the remaining 400 were synthetic images generated from the observed crack-pattern characteristics. Although Gaussian-noise regularization, Wasserstein loss, and unpaired training reduce mode collapse, the model may remain biased toward the morphology distribution represented by these data. Consequently, the current experiments do not establish generalization to different pavement structures, crack types, moisture conditions, GPR systems, or regional material characteristics.

The primary cause of mode collapse lies in the similar probability density distribution of the input and the limited generalizability of the samples, which lead the generator to produce only a small number of image variants, thereby failing to capture the diversity inherent in the true data distribution. To address this issue, random Gaussian noise was introduced as a disturbance term during the training phase to slightly disturb the input B-scan, thereby altering their underlying probability distributions. The incorporation of Gaussian noise enhances the model’s robustness and responsiveness to input variability, encouraging the exploration of more diverse mapping patterns. Figure 22 provides a visual demonstration of the effects of Gaussian noise injection: the generated cracks are no longer restricted to the central region of the image but vary in position in accordance with the spatial location of hyperbolic anomalies in the input. Additionally, the crack morphologies, orientations, and spatial distributions differ significantly across samples, preliminarily reflecting the structural diversity observed in real cracks.

From a quantitative perspective, the U-Net CycleGAN with noise injection achieves further improvements, with increases of 0.0674 in MS-SSIM, 0.2856 in FID, and 8.6797 in LPIPS, indicating that the generated images are more consistent with the target domain. Furthermore, the crack morphologies generated by U-Net CycleGAN are qualitatively superior to those of the original CycleGAN. As shown in Figure 22, the cracks produced by U-Net CycleGAN exhibit greater complexity, with large-angle extension patterns more closely resembling the real crack morphologies observed in CT scans. In contrast, cracks generated by the original CycleGAN are predominantly spring-like and vertically aligned, with uniform directional patterns largely concentrated in the center of the image, as highlighted by the black boxes in Figure 20. Despite the observed improvements in crack position and morphological realism, the current model still struggles to reconstruct opening width and depth, thereby falling short of fully satisfying the objectives of this study concerning crack morphology inversion.

The introduction of crack-dimension-constrained cycle-consistency loss incorporates crack dimension labels into the training process, thereby providing the model with prior knowledge and physical constraints. It guides the network toward generating images that are more consistent with the actual geometrical dimensions of structural cracks. As illustrated in Figure 22, the generated images exhibit strong agreement with the original labels in terms of upper and lower crack opening widths, crack depths, and spatial positioning, validating the model’s ability to recover crack morphology and control geometrical dimensions. The effectiveness of this loss function is further supported by the quantitative results in Table 4. Compared to the U-Net CycleGAN model without labels, the crack-dimension-constrained cycle-consistency model achieves superior scores across multiple evaluation metrics, with MS-SSIM, LPIPS, and FID reaching 0.9731, 0.1141, and 3.6234, respectively. Moreover, the NRMSE for cycle consistency improves by 0.0147, indicating more accurate structural inversion during the cyclic generation process.

However, the strong constraints introduced by the crack-dimension-constrained cycle-consistency loss also give rise to certain limitations. As observed in the generated outputs, the crack patterns tend to be overly smooth, particularly at regions of directional change, where unnatural curvature transitions occur. These smooth transitions fail to capture non-smooth morphological features such as fractures and sharp corners, which are characteristic of real crack formation processes. Such artifacts are evident in the blue-boxed region of Figure 20. Additionally, blurred edges and discontinuities at the interface between the surface and base layers, as highlighted in the red-boxed region of Figure 20. These deficiencies may be attributed to the model prioritizing label conformity at the expense of natural morphological variation.

To address the limitations introduced by crack-dimension-constrained cycle-consistency loss, Wasserstein Loss was further incorporated to improve the generation quality of the model. Unlike traditional adversarial losses based on JS divergence or KL divergence, Wasserstein Loss offers a more relaxed yet stable optimization objective, allowing the generator to generate diverse solutions within the target image domain more naturally and continuously. This leads to a generated distribution that more closely aligns with the style and global structure of real samples. As shown in Figure 20, the introduction of Wasserstein Loss enables more extensive crack propagation, particularly in regions where the crack pattern exhibits directional changes. These transitions appear more natural and better reflect the realistic development law of structural cracks, thereby avoiding the overfitting caused by previously imposed strong constraints. Additionally, background noise in non-crack areas is significantly reduced, and edges become clearer and more coherent, as highlighted in the green boxed region of Figure 20.

As reported in Table 4, the model with Wasserstein Loss achieved further improvements across all evaluation metrics, attaining final scores of 0.9984 for MS-SSIM, 0.0566 for LPIPS, and 2.3145 for FID. These results indicate that the generated images not only approximate the target domain more closely but also represent the geometrical dimension of cracks more accurately. Collectively, these findings suggest that Wasserstein Loss enhances overall image quality and maintains label consistency while resolving the unnatural morphological constraints. Ultimately, by integrating four key components—U-Net generator, Gaussian noise injection, crack-dimension-constrained cycle-consistency loss, and Wasserstein loss—a complete GPR-CMIGAN model was constructed, achieving enhanced morphological realism and generative stability in the task of crack image inversion.

## 5. Conclusions

In this study, a method GPR-GDMI, is proposed for the detection of structural crack dimensions and the morphology inversion of structural cracks in semi-rigid asphalt pavements, driven by field-collected GPR B-Scans and real crack morphology data. The following conclusions can be drawn.

(1) The proposed GPR-GDMI was developed based on field-collected GPR, enabling the detection of structural crack geometrical dimensions and the inversion of crack morphology.

(2) The application of DCA in GPR-GCDNet effectively enhanced the network’s ability to focus on critical regions, while the multi-scale fusion strategy expanded the receptive field, contributing positively to the detection of crack geometrical dimensions.

(3) On the 70-sample GPR-GCDNet test subset, which was constructed through source-level parti-tioning by physical crack and survey section, GPR-GCDNet achieved an AP of 0.84, an F1-score of 0.89, and an mIoU of 0.74 under an IoU threshold of 0.5, indicating reliable performance in de-tecting the dimensional characteristics of cracks from GPR B-scan images.

(4) In GPR-CMIGAN, the injection of random Gaussian noise effectively mitigated the problem of mode collapse. The integration of a crack-dimension-constrained cycle-consistency principle and Wasserstein loss enabled the generated crack morphology to maintain consistency with the upper and lower opening widths and depth as predicted by GPR-GCDNet.

(5) Future research will focus on addressing the current limitations of the GPR-GDMI framework, such as the limited transferability of GPR-GCDNet to other types of distress detection and its optimizable detection efficiency, as well as the lack of physical interpretability in the mid-section of the inversion crack morphology produced by GPR-CMIGAN.

## Figures and Tables

**Table 1:** Dataset splitting in GPR-GCDNet and GPR-CMIGAN.

Network	Train	Val	Test	Total	Split Ratio
GPR-GCDNet	493	141	70	704	7:2:1
GPR-CMIGAN (B-Scan)	414	\	46	460	9:1
GPR-CMIGAN (CT Scan)	414	\	46	460	

**Table 2:** Result of Hyperparameter Ablation Experiment.

Model	AP	AP Increasing	F1	F1 Increasing	mIoU	mIoU Increasing	FLOPs	FLOPs Increasing
Layer = 2	0.28	-	0.36	-	0.39	-	40.48 G	-
Layer = 4	0.58	105.6%	0.67	85.6%	0.53	36.8%	41.02 G	1.3%
Layer = 6	0.72	23.3%	0.82	22.5%	0.66	24.6%	42.81 G	4.4%
Layer = 8	0.84	16.7%	0.89	8.4%	0.74	12.0%	45.67 G	6.7%
Layer = 10	0.89	6.3%	0.93	4.8%	0.77	3.8%	50.42 G	10.4%

**Table 3:** Result of the GPR-GCDNet Comparison Experiment.

Model	Params	AP	F1	mIoU	FLOPs	FPS
	M	\	\	\	G	Frames/Second
Ours	66.51	0.84	0.89	0.74	45.67	28.42
CSPDarket	65.53	0.81	0.85	0.69	65.53	46.31
ResNet101	69.36	0.67	0.72	0.55	21.11	22.41
Mobilenetv4	68.99	0.77	0.81	0.64	12.62	62.55
EfficientNet-B4	45.51	0.79	0.82	0.70	24.63	43.25
ShuffleNet	28.89	0.70	0.76	0.60	21.52	73.63

**Table 4:** Result of the GPR-CMIGAN Comparison Experiment.

Model	MS-SSIM	LPIPS	FID	NRMSE	PSNR
Cyclegan	0.8168	0.5930	18.5123	0.5243	11.5194
U-Net	0.8904	0.4731	14.3622	0.3098	26.2241
Cyclegan + Noise	0.9475	0.2241	7.5414	0.1837	34.7152
U-Net + Noise	0.9578	0.1875	5.6825	0.1483	38.5625
U-Net + Noise +Crack-dimension-constrained cycle-consistency loss	0.9731	0.1141	3.6234	0.0147	47.6743
GPR-TransUNet [25]	0.9832	0.1032	3.2156	0.0133	54.3214
MHInvNet [47]	0.9865	0.0832	3.0526	0.0123	56.1367
MSInv-GPR [48]	0.9912	0.0947	2.8341	0.0112	59.7823
DGPRI-Net [49]	0.9907	0.0732	2.5632	0.0090	61.4627
Ours	0.9984	0.0566	2.3145	0.0088	62.4922

## Data Availability

The raw data supporting the conclusions of this article will be made available by the authors on request.
